# Molecular Evolution of *Drosophila* Cuticular Protein Genes

**DOI:** 10.1371/journal.pone.0008345

**Published:** 2009-12-17

**Authors:** R. Scott Cornman

**Affiliations:** Department of Cellular Biology, University of Georgia, Athens, Georgia, United States of America; Institut de Genetique et Microbiologie, France

## Abstract

Several multigene families have been described that together encode scores of structural cuticular proteins in *Drosophila*, although the functional significance of this diversity remains to be explored. Here I investigate the evolutionary histories of several multigene families (CPR, Tweedle, CPLCG, and CPF/CPFL) that vary in age, size, and sequence complexity, using sequenced *Drosophila* genomes and mosquito outgroups. My objective is to describe the rates and mechanisms of ‘cuticle-ome’ divergence, in order to identify conserved and rapidly evolving elements. I also investigate potential examples of interlocus gene conversion and concerted evolution within these families during *Drosophila* evolution. The absolute rate of change in gene number (per million years) is an order of magnitude lower for cuticular protein families within *Drosophila* than it is among *Drosophila* and the two mosquito taxa, implying that major transitions in the cuticle proteome have occurred at higher taxonomic levels. Several hotspots of intergenic conversion and/or gene turnover were identified, e.g. some gene pairs have independently undergone intergenic conversion within different lineages. Some gene conversion hotspots were characterized by conversion tracts initiating near nucleotide repeats within coding regions, and similar repeats were found within concertedly evolving cuticular protein genes in *Anopheles gambiae*. Rates of amino-acid substitution were generally severalfold higher along the branch connecting the Sophophora and Drosophila species groups, and 13 genes have Ka/Ks significantly greater than one along this branch, indicating adaptive divergence. Insect cuticular proteins appear to be a source of adaptive evolution within genera and, at higher taxonomic levels, subject to periods of gene-family expansion and contraction followed by quiescence. However, this relative stasis is belied by hotspots of molecular evolution, particularly concerted evolution, during the diversification of *Drosophila*. The prominent association between interlocus gene conversion and repeats within the coding sequence of interacting genes suggests that the latter promote strand exchange.

## Introduction

Arthropod cuticle is an important ecological innovation of a highly successful invertebrate phylum. It is a strong, light exoskeleton and environmental interface that is predominantly composed of an ordered matrix of chitin fibers and protein [1]. The biomechanical properties of cuticle and the control of its deposition during development have long been of interest. More recently, evidence has emerged that cuticular proteins are relevant to problems in applied entomology, such as the adaptation of disease vectors to human-associated selective pressures [2], [3], [4]. Cuticular proteins are further of interest because of the high level of gene conversion occurring among certain genes in some insect species [5], [6], [7], [8].

Recent studies have revealed a remarkable diversity of cuticular proteins. By far the largest, and taxonomically most widespread, cuticular protein family is the CPR family, which is characterized by a conserved domain first identified by Rebers and Riddiford [9]. This domain has been revised and extended in the genomic era but is still commonly referred to as the “R&R Consensus”. Examples of this domain have been shown to bind chitin *in vitro* [10], [11]. Additional cuticular protein families have been uncovered by methods such as mutant analysis and shotgun proteomics. Some families appear to be narrowly restricted taxonomically (e.g., the apidermin [12] and CPLCW [7] families) and presumably are of more recent origin. Other families that are widely distributed are nonetheless characterized by radiations within particular taxa [6], [7]. Thus, the complement of cuticular proteins is dynamic among insect orders, but the pace of change has not been investigated and the functional significance of these gene expansions is unknown.

While functional studies can shed light on the roles of specific proteins during development, an integrated view of how the molecular complexity of cuticle has evolved remains a daunting task for its sheer scale. For example, are major transitions in the cuticular proteome associated with changes in ecology and/or development, and at what taxonomic scale? As they become established in the molecular repertoire, do duplicated genes adaptively differentiate at the protein sequence level, or are differences primarily regulatory? Are some protein families more conserved than others, either at the level of protein sequence, gene regulation, or copy number? An important foothold in this genomic landscape can be gained by identifying genomic patterns of gene gain and loss, and by measuring substitution rates among orthologous sequences. This evolutionary perspective provides a context for planning and interpreting comparative functional studies that have broadest impact.

In this paper, I present an evolutionary analysis of cuticular protein families of *Drosophila*, using the mosquitoes *Anopheles gambiae* and *Aedes aegypti* as sister groups, because of the genomic sequence data and gene-family annotations that are available. I compare evolutionary rates of change in gene number across gene families and across taxa, and examine the organization of these gene families in the genome. The questions I address with these data are: How has the number and organization of genes in each gene family changed in a phylogenetic context? Do some gene families or subfamilies change in size more rapidly than others? Has intergenic gene conversion, which has been reported for some cuticular proteins in some species [5], [8], occurred more broadly in the evolutionary history of *Drosophila* cuticle genes? Are certain genes particularly susceptible to such events? Finally, I compare orthologous sequences to investigate whether there has been any general change in the rate of evolution of cuticular proteins along the *Drosophila* phylogeny. In particular, is there evidence of positive selection driving divergence of protein sequence in a particular lineage? If so, it would imply that cuticular proteins participate in the adaptive divergence of species.

## Methods

Using published gene family annotations as a seed for BLAST, I searched seven *Drosophila* taxa and the mosquitoes *An. gambiae* and *Ae. aegypti* for genes of the CPR [13], Tweedle [14], CPF/CPFL [15], [16], and CPLCG [7] families. These are the best characterized multigene families that encode structural cuticular proteins. I excluded other known *Drosophila* cuticular protein ‘families’ that consist of only one or two genes and are not also present in mosquitoes (e.g. *Edg91* [17]). I also excluded homologs of putative cuticular proteins identified in *An. gambiae* (the CPLCA and CPLCP families [7]) that are not yet well characterized in *Drosophila*.

The annotation of *An. gambiae* cuticular proteins has been described elsewhere [6], [7], [16]. *Ae. aegypti* genes were identified by BLAST as well as by searching Ensembl (http://www.ensembl.org) gene annotations. The *Drosophila* annotations were based primarily on previous studies [13], [14], [16] but were amended if necessary based on ortholog alignments. Particularly noteworthy is the inclusion of an additional CPR gene, *CG13670*, upstream of the genes *Cpr66Ca* and *Cpr66Cb* that were annotated by [13].

**Figure 1:**
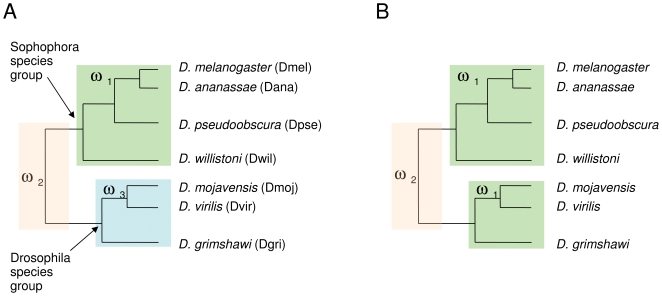
Schematic representing the estimation of ω, the ratio of nonsynonymous to synonymous substitutions, along branches of the *Drosophila* phylogeny. A. Based on initial pairwise calculations of Ka/Ks, three distinct ω parameters were estimated for each set of orthologous genes. One ω was estimated for the Sophophora species group, one for the Drosophila species group, and a third ω was estimated for the branch connecting the two species groups. The branches of the tree labeled with each ω class are indicated by colored boxes. B. To test whether the estimated ω for the branch between the Sophophora and Drosophila groups is significantly greater than 1 for a particular set of orthologous genes, ω is recalculated for that branch with all other branches assigned to a single background ω class.

I annotated only six other *Drosophila* genomes in addition to *D. melanogaster* (*D. annasae*, *D. pseudoobscura*, *D. willistoni*, *D. mojavensis*, *D. virilis*, and *D. grimshawi*) because this number well represents the phylogenetic diversity of sequenced *Drosophila* (**Fig. 1**). Coding sequences of these *Drosophila* species were predicted from genomic regions containing BLAST matches using the programs Genscan [18] and SNAP [19], and manually adjusted based on ortholog alignments. This approach serves to identify the number of genes and the coding sequence of the mature predicted proteins (that is, after signal peptide cleavage). It is not intended to serve as a complete annotation strategy, which would be redundant to ongoing efforts by other groups (see http://rana.lbl.gov/drosophila/index.html).

I did not exhaustively annotate the other five sequenced *Drosophila* genomes because they are closely related to taxa already represented and would provide little additional information while substantially increasing the computational requirements for the analyses. However, I did annotate particular genes in these species when necessary to clarify recurring patterns of intergenic conversion (see Results). Annotated pseudogenes in *D. melanogaster* and the presumed orthologous sequence in other species are noted in some figures but were not included in total gene counts.

All sequences used in this study are given in **Text S1** and inferred orthology is given in **Text S2**. Neighbor-joining trees were created with MEGA3 [20] using Clustal-aligned amino acid sequence, the amino-acid exchange matrix of Jones et al. [21], and pairwise deletion of indels. Bootstrap values represent 1,000 resampled data sets.

Several methods have been described that assess the tempo of gene family evolution. Here I use the CAFÉ program [22], which parameterizes the rate of gene gain or loss along an organismal phylogeny by fitting a model to the observed distribution of genes in each species and inferring the number of genes at ancestral nodes. The rate is expressed in absolute time assuming independent estimates of divergence are available. The *Drosophila* phylogeny and branch lengths (in absolute time) follow [23]. The divergence time between *An. gambiae* and *Ae. aegypti* is assumed to be 95 million years based on [24], and 260 million years between *An. gambiae* and *D. melanogaster* based on [25].

To evaluate patterns of duplication and/or gene conversion, I used the program Dotter [26] to create dot plots of tandem arrays and the program RDP2 [27] to estimate the probability of gene conversion events. The dot plots shown are graphical depictions of genomic sequence aligned with itself, in which the presence of a dot at any X-Y coordinate indicates nucleotide similarity between sliding windows centered on positions X and Y of the sequence [26]. The dot plots shown in the Results depict patterns of similarity within both the input sequence and its reverse complement, and there is always a dot at positions along the diagonal (X = Y) by definition. Substitutions per site for pairwise comparisons were calculated with DnaSP [28]. I used PAML [29] to estimate Ka/Ks along branches of gene trees. PAML uses a likelihood framework to estimate a parameterization of Ka/Ks, termed ω, to model sequence evolution. This type of branch-specific analysis is appropriate for estimating rates of ortholog diversification provided that synonymous sites are not saturated. For all analyses, the ratio of transitions to transversions (κ) was estimated in order to assess mutational saturation. All multiple sequence alignments were performed with ClustalW using default protein parameters, but were also manually inspected and trimmed of ambiguously aligned regions or regions likely to be concertedly evolving.

Because of the large number of parameter-rich tests of sequence evolution that can conceivably be performed on a given alignment, it is important to clearly articulate the tested hypotheses and use a hierarchical approach to data analysis that allows suitable likelihood tests. The evolutionary models used in PAML are stated for each analysis in the Results.

## Results

### Gene-Family Size Evolution

The number of annotated genes of each cuticular protein family varies substantially among *Anopheles*, *Aedes*, and *Drosophila* (**Table 1**). In contrast, the number of genes in the seven *Drosophila* genomes I investigated is very similar (**Table 2**), implying a low rate of gene turnover within the genus. I quantified rates of change in the total number of cuticular proteins using the CAFÉ program [22]. This method estimates a birth-and-death parameter, λ, defined as the change in gene number per million years. By this measure, the rate of change in the number of cuticular proteins is roughly an order of magnitude lower within *Drosophila* (3E-04) than it is among the three Dipteran genera (3.8E-03). Although λ = 3.8E-03 genes/10^6^ years was the most likely rate for the latter group, even higher parameter values were possible but could not be evaluated because of a methodological constraint [22] requiring that the product of λ and the longest branch length not exceed one.

**Table 1:** Number of genes of four cuticular protein gene families in three Dipteran insect genomes.

Species	CPR	CPLCG	CPF/CPFL	Tweedle
*Drosophila melanogaster*	102	3	3	27
*Anopheles gambiae*	156	27	11	12
*Aedes aegypti*	240^1^	16	12	9

1 Number of Ensembl-annotated proteins (v. 40) with the Rebers and Riddiford Consensus (CPR family consensus domain, Pfam00379), which should closely approximate the total number of CPR genes.

**Table 2:** Number of genes of each gene family in seven *Drosophila* species.

Species	CPR	CPLCG	CPF/CPFL	Tweedle
*D. melanogaster*	102	3	3	27
*D. ananassae*	104	3	3	27
*D. pseudoobscura*	101	3	3	30
*D. willistoni*	103	3	3	28
*D. virilis*	100	3	3	26
*D. mojavensis*	104	3	3	26
*D. grimshawi*	100	3	3	27
Reconstructed ancestral state	102	3	3	27

*Drosophila* CPR genes often occur on chromosomes as ‘singleton’ genes, linearly distant from other homologs. However, most (∼75%) are organized into tandem arrays of genes, with most or all of the genes in the array clustering together phylogenetically (see [13] and **Text S3**). These arrays are likely to include monophyletic groups that have arisen through tandem duplication from an ancestral gene. Because of the short lengths of paralogous alignments and overall conservation of the defining domain of CPR proteins, bootstrap support for the monophyly of these groups is usually low. Nonetheless, given their physical arrangement and phylogenetic clustering, it is reasonable to treat these arrays as subgroups (**Table 3**) for further analysis (all CPR subgroups discussed here are referenced by *D. melanogaster* chromosome band, see [13]).

**Table 3:** Number of genes within Drosophila CPR tandem arrays.

Lineage	30F	44C*	47E	49A	62B	64A	65A*	65E	66C	67F^1^	72E	76B	78C	84A	97E
*D. melanogaster*	2	4	7	8	3	4	18	3	3	3	3	4	2	8^2^	2
*D. ananassae*	2	4	7	8	3	4	19	3	3	3	3	4	2	8^2^	2
*D. pseudoobscura*	2	4	7	8	3	4	16	4	3	2	3	4	2	7	2
*D. willistoni*	2	5	7	7	3	4	18	3	3	2	3	3	2	8	2
*D. virilis*	2	5	7	8	3	4	15	3	3	2	3	4	2	8	2
*D. mojavensis*	2	9	7	8	2	4	15	3	3	3	3	4	2	8	2
*D. grimshawi*	2	2	8	8	3	4	16	3	3	2	3	5	2	8	3
Reconstructed ancestral state	2	4	7	8	3	4	18	3	3	2	3	4	2	8	2

* Rate of change in gene number significantly different from assigned λ = 3.8E-4 at P<0.05.

1 Found significant at P<0.01; however the state reconstructed by CAFE for the *D. melanogaster–D. ananassae* node was 2 rather than 3, thereby doubling the number of inferred changes. BLAST searches of the melanogaster-group species *D. erecta*, *D. yakuba*, *D. simulans*, and *D. sechellia* reveal three genes in each species. Running CAFÉ with these additional data for this subfamily resulted in a reconstructed ancestral state of three genes and no significant deviation from the overall λ.

2 Includes *DmelCpr5C* and its ortholog in *D. ananassae*, which I infer to be an interchromosomal duplication of *Ccp84Ac* (see Text S2).

I first used CAFÉ to test whether, collectively, tandemly arrayed or singleton CPR genes evolved differently from the overall rate of change in gene number. Neither category deviated statistically from the rate of λ = 3.0E-04 calculated from the data for all gene families in **Table 2**. This result is somewhat surprising given that tandemly arrayed genes might be expected to have a higher rate of gene duplication than singleton genes, due to unequal crossing over among homologous sequences. However, two individual arrays, the 44C and 65A arrays, did differ significantly (P<0.05) from the overall λ when considered separately. Note that the small array consisting of *Cpr50Ca* and *Cpr50Cb* was excluded from this analysis because the former contains a known non-cuticular protein gene (*CG13340*) within an intron.

**Figure 2:**
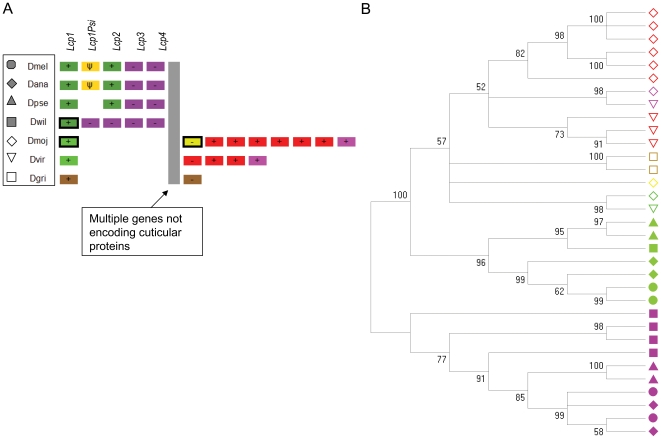
The array of CPR cuticular protein genes located approximately at band 44C of *D. melanogaster* chromosome 2R and the orthologous regions in six other *Drosophila* species. A. Schematic of the organization of genes in the array, with colored boxes matching colored symbols in the phylogeny according to the legend at left. Names at top are of *D. melanogaster* genes; plus and minus symbols indicate relative orientation. Genes with dark outlines are predicted to be intronless. B. Neighbor-joining phylogeny (see Methods) of predicted amino acid sequence with bootstrap support indicated.

**Figure 3:**
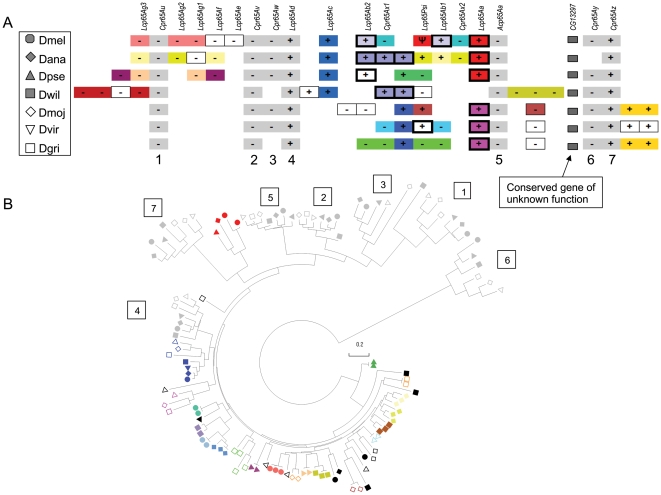
The array of CPR cuticular protein genes located approximately at band 65A of *D. melanogaster* chromosome 3L and the orthologous regions in six other *Drosophila* species. A. Schematic of the organization of genes in the array, with colored boxes matching colored symbols in the phylogeny according to the legend at left. Numbered positions in the array correspond to numbered clades in the phylogeny of part B. Names at top are of *D. melanogaster* genes; plus and minus symbols indicate relative orientation. B. Neighbor-joining phylogeny (see Methods) of predicted amino acid sequence. Arrow indicates the clade within which genes cluster as paralogs rather than as orthologs (see text for details). Bootstrap support is not shown for clarity, but is greater than 90% for all numbered groups and generally low outside these groups.

The organization of the 44C array in the seven *Drosophila* species is shown in **Figure 2A** and a phylogeny of these genes is shown in **Figure 2B**. The organization and phylogenetic relationships of the 65A array are shown in **Figure 3A** and **Figure 3B**, respectively. These figures show that gene gain and loss within the 44C and 65A arrays is likely greater than suggested by total gene number alone. While the gene trees of other CPR subfamilies show a clear pattern of conserved one-to-one orthology with infrequent gene gain or loss (**Text S3**), the pattern is strikingly different for these two arrays. In the 44C array, genes generally cluster by species in one or two distinct groups. Moreover, all genes in the Drosophila species group are more closely related to each other than to any gene in the Sophophora species group, and vice versa. The presence of closely related paralogs within each lineage could, in principle, be due to high rates of gene duplication and loss within this array. However, a previous study of polymorphism at coding and flanking sites in the *D. melanogaster* 44C array [8] indicates a contribution by interlocus gene conversion as well.

The 65A array includes seven genes that are present in most or all species as one-to-one orthologs, in a conserved order. In the gene tree, these ortholog sets all have bootstrap support greater than 90%. Other sets of genes also occur as one-to-one orthologs but are limited to either the Drosophila or Sophophora species groups and presumably arose after the divergence of these clades. A third category includes sets of genes that cluster as paralogs within each lineage, similar to that observed in the 44C array. Some of these sets of genes are adjacent whereas others are not, and where they occur in the array is quite variable. Genes in this third category are more closely related to each other across *Drosophila* species than they are to the conserved single-copy genes. This result suggests that a single ancestral gene, or a few closely related genes, gave rise to an eclectic set of descendants within each lineage, due to a higher rate of gene turnover, gene conversion, and/or gene rearrangement than other genes in the same array. Charles et al. [5] previously presented evidence for tandem duplications and intergenic conversion in the evolution of the *D. melanogaster* 65A array, and even detected variation in gene copy number among different strains of that species. The multi-species, whole-genome analysis presented here (**Fig. 3B**) reveals that these processes are constrained to a particular region of the gene tree, outside of which genes show one-to-one patterns of homology and synteny among species.

Given that the 44C and 65A arrays evolve more rapidly and contain sets of similar paralogs, it is of interest to determine if retrogene formation is an important contributor to their evolutionary history. Retrogenes are suggested by the absence of introns present in homologs, divergent or missing promoter sequence, and 3′ thymine runs. Indeed, a modest number of gene predictions within the 44C and 65A arrays lack introns, indicated by thick borders in **Figures 2A** and **3A**, and sites where intronless genes occur in the array are consistent across species. Intronless gene models were manually checked to verify that a valid signal peptide was predicted. In a few cases, candidate TATA boxes were not identified; however, some CPR genes in *An. gambiae* that are detectably expressed also lack TATA boxes [30]. Furthermore, polyT runs were not evident downstream of coding sequences. Thus, the intronless genes in these arrays are more likely to have arisen through ectopic recombination or splice-site mutations. (Note that most CPR genes contain a single intron placed after the first few codons of the signal peptide, and that evidence of intron-regulated gene expression has been found for *Acp65A* in *D. melanogaster* [31]). Even if all of the intronless genes in these arrays actually arose through retroposition, the presence of highly similar paralogs is not limited to such genes, nor do all intronless genes cluster with within-species paralogs. Thus, retrogenes do not appear to contribute substantially to the distinctive pattern of evolution within these arrays.

In summary, comparing the 44C and 65A arrays to the rest of the *Drosophila* CPR gene family, I conclude 1) that the greater variation in gene number detected by CAFÉ within these arrays is associated with paralog homogenization; and 2) that there can be pronounced biases as to which genes within arrays are susceptible to sequence homogenization.

### Intergenic Conversion Recurring between Specific Gene Pairs

**Figure 4:**
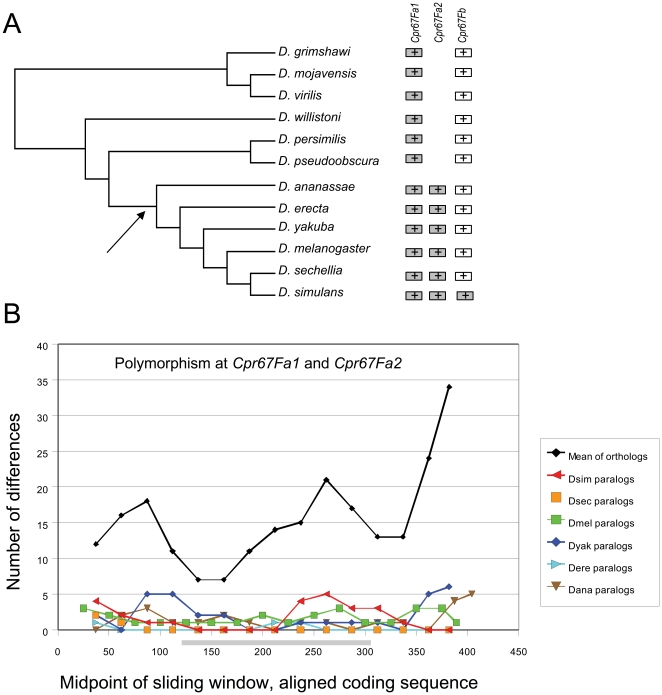
Gene organization and polymorphism patterns within the 67F array of *Drosophila* CPR genes. A. Gene organization in the 12 *Drosophila* genomes. The arrow indicates the *melanogaster* species group. Gray boxes indicate highly similar paralogs. B. Number of polymorphic sites within a sliding window of 50 bases across aligned *Cpr67Fa1* and *Cpr67Fa2* alleles (step of 25 bases). Polymorphism between paralogs is much lower than between orthologs across the entire coding region. The part of the X axis corresponding to the R&R Consensus is shaded.

To clarify whether the presence of very similar paralogs is distinctive of the 44C and 65A arrays, I systematically examined phylogenetic patterns within all other CPR arrays in *Drosophila* (**Text S3**). Two additional arrays at 67F and 84A showed pronounced clustering among the seven genomes initially examined. I then examined these genes in the remaining genomes to determine the consistency of paralog clustering across species. Consistent clustering of paralogs and significant evidence of intergenic conversion was identified within the 67F tandem array for genes *Cpr67Fa1* and *Cpr67Fa2*, but only within the *melanogaster* species group (**Figure 4A**) and not between either gene and *Cpr67Fb*. Note that in *D. simulans*, there are three *Cpr67Fa*-like genes and *Cpr67Fb* is absent, but dot plots reveal the intergenic sequences to be nearly identical as well (not shown). This latter finding suggests that the three gene copies are the result of recent tandem duplication within this lineage rather than intergenic conversion. Only one *Cpr67Fa*-like gene is present in the other *Drosophila* species.

**Figure 4B** illustrates the number of nucleotide differences between paralogous *Cpr67Fa*-like genes in the *melanogaster* species group compared with among-ortholog variation. I used a sliding window of 50 bp to measure variation in 25-bp steps along the length of the aligned coding sequence (note that because gaps are excluded, the length of the region compared varies among alignments). There are many fewer nucleotide differences between paralogs than among orthologs, which is unexpected given the implicitly older age of paralogs. These data in conjunction with the phylogenetic pattern strongly imply concerted evolution at these loci.

A formal test of gene conversion between the sequenced alleles of *Cpr67Fa1* and *Cpr67Fa2* was done with the RDP2 program. For this test, I initially aligned the region extending 500 bp upstream of the start of the second exon to 500 bp downstream of the stop codon. At the 5′ end, this region includes the short intron within the signal peptide, the first exon, and sequence upstream of the predicted promoter. The polyadenylation signal was also included at the 3′ end. I then manually trimmed the alignment edges to remove regions that were too divergent to provide phylogenetic signal. For all species in the *melanogaster* species group, absence of gene conversion between paralog pairs was rejected by RDP2 at P<0.001.

**Figure 5:**
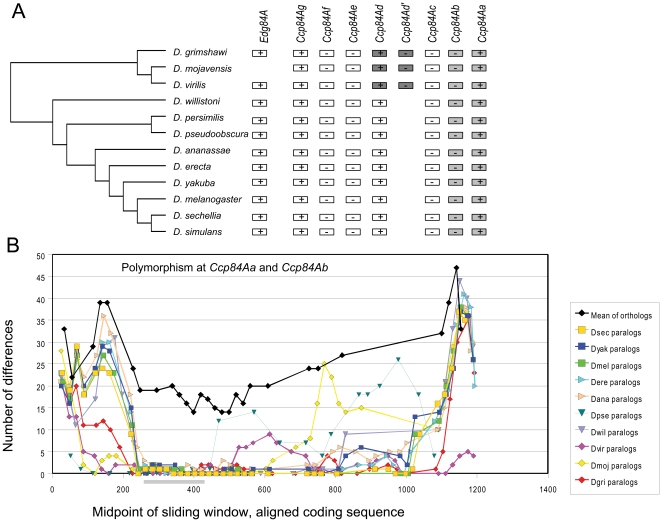
Gene organization and divergence patterns within the 84A array of *Drosophila* CPR genes. Note that genes in this array have the historical names *Edg84A* and *Ccp84Aa-g* and thus diverge from nomenclature for other arrays. A. Gene organization in ten *Drosophila* genomes. *D. persimilis* and *D. pseudoobscura* were excluded due to the presence of gaps in the genome sequence. Pairs of genes that cluster by species rather than with orthologs are shaded corresponding shades of gray. B. Graph of the number of polymorphic sites within a sliding window of 50 bases between aligned *Ccp84Aa* and *Ccp84Ab* alleles (step of 25 bases). Polymorphism is much lower between paralogs than between orthologs in the central region of the gene, which includes the R&R Consensus, but tends to increase at the 5′ and 3′ ends of coding sequence, approaching the level of orthologs. The part of the X axis corresponding to the R&R Consensus is shaded.

Within the 84A array, the genes *Ccp84Aa* and *Ccp84Ab* (**Fig. 5A**) clustered together for all species for which there were complete genomic sequence, although there are few differences to distinguish *D. simulans* and *D. sechellia* alleles at these loci. Sequence polymorphism among paralogous and orthologous sequences is shown in **Figure 5B** (note that the *D. simulans* and *D. persimilis* genomes has gaps in this region and were excluded). Within the coding sequence, particularly around the R&R Consensus, there is again very low polymorphism among paralogs compared with the mean among orthologs. However, unlike the 67F example, the polymorphism among paralogs is high at the 5′ and 3′ ends of the coding sequence, approaching the level observed among orthologs. Thus, concerted evolution has primarily homogenized the region around the R&R Consensus domain. Tests of gene conversion between *Ccp84Aa* and *Ccp84Ab* performed with RDP2 were significant at P<0.001 for all species.

**Figure 6:**
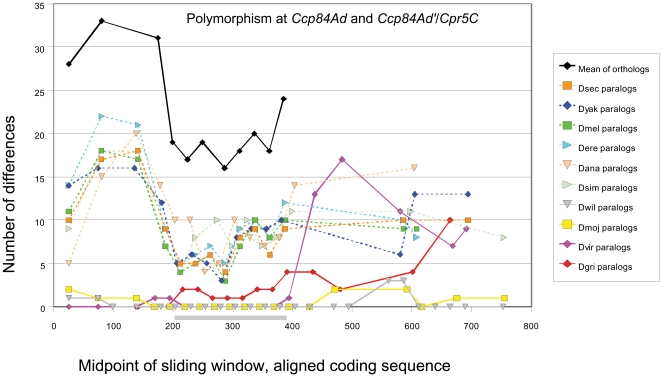
Graph of the number of polymorphic sites within a sliding window of 50 bases between aligned *Ccp84Ad* and *Ccp84Ad′/Cpr5C* alleles (step of 25 bases). Note that *Cpr5C* is the presumed ortholog of *Ccp84Ad′* based on amino-acid sequence phylogeny, but occurs on the X chromosome (see text). Polymorphism is lower between paralogs than between orthologs for all comparisons. However, the level of paralog polymorphism is lowest for those species (solid lines) that have *Ccp84Ad′*. Species (dotted lines) that lack *Ccp84Ad′* but instead have *Cpr5C* show intermediate levels of divergence. The part of the X axis corresponding to the R&R Consensus is shaded.

Also within the 84A array, the gene *Ccp84Ad* and an adjacent gene (referred to here as *Ccp84Ad′*) present in four species (the *Drosophila* group plus *D. willistoni*) exhibit a pattern of within-species clustering similar to that of *Ccp84Aa* and *Ccp84Ab* (**Figure 5A**). In *D. melanogaster* and *D. ananassae*, there is no gene between *Ccp84Ac* and *Ccp84Ad*. Rather, the orthologous sequence appears by phylogeny to be *Cpr5C*, which is on the X chromosome. In *D. pseudoobscura*, the orthologous gene appears to have been lost. Divergence between the paralogs *Ccp84Ad* and *Ccp84Ad′* is much lower than it is among orthologs, as was seen for the *Ccp84Aa/Ccp84Ab* and *67Fa1/67Fa2* comparisons. Significant evidence of gene conversion (P<0.001) was found for all four species that contain both *Ccp84Ad* and *Ccp84Ad′*. Interestingly, in those species that have a *Cpr5C*-like gene (i.e., a gene most similar to *Cpr5C* of *D. melanogaster* and that is not in the 84A array but rather elsewhere in the genome), the level of divergence between it and *Ccp84Ad* is intermediate between that of *Ccp84Ad/Ccp84Ad′* and all *Ccp84Ad* orthologs (dotted lines in **Fig. 6**). The sliding-window plot shows a 5′ to 3′ pattern of divergence between *Cpr5C* and *Ccp84Ad* in these species that is strikingly similar in form to that of the *Ccp84Ad* orthologs, reaching its lowest levels within the R&R Consensus. This pattern suggests an earlier period of low paralog divergence followed by a resumption of independent evolution. That is, gene conversion likely occurred between *Ccp84Ad* and *Cpr5C* prior to the movement of this gene from the 84A array to the X chromosome (as is implied by species phylogeny). Thus, the loss of one of the two interacting genes from the array has terminated the pattern of concerted evolution within those lineages, rather than being replaced by a new interacting pair of paralogs.

Taken together, the patterns within the 67F and 84A arrays present a contrasting type of evolutionary ‘hotspot’ compared with the 65A and 44C arrays. In the former, recurring gene conversion between specific gene pairs, rather than gene turnover, underlies concerted evolution of paralogs. In the latter two arrays, I did not find evidence of extensive intergenic conversion between paralogs within these arrays (results not shown). Gene turnover under a birth-and-death model [32], which can result in the loss and replacement of older gene duplicates with younger ones, is therefore the more likely cause of sequence similarity among paralogs in these regions.

**Figure 7:**
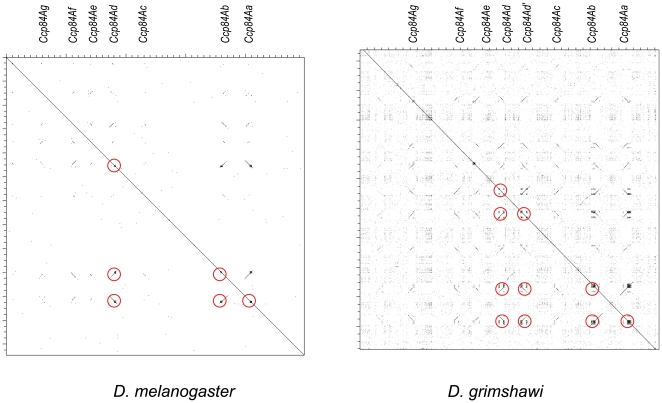
Dot plots of the 84A array in two *Drosophila* species that illustrate sequence repetition (indicated by red circles) within the coding regions of those genes evolving concertedly. Independently evolving genes in the array lack this sequence repetition. Dot plots of this region for the other ten *Drosophila* species are shown in Text S4.

I used Dotter [26] to create dot plots of the 84A arrays of all twelve *Drosophila* species, to further explore patterns of sequence similarity as well as identify any unusual sequence features of paralog pairs undergoing concerted evolution. The dot plots of *D. melanogaster* and *D. grimshawi* are shown in **Figure 7** as examples; the remaining dot plots are shown in **Text S4**. From these dot plots I identified an association between the presence of nucleotide repetition within the coding region and gene pairs undergoing concerted evolution. *Ccp84Aa* and *Ccp84Ab* have this repetitive sequence, as do *Ccp84Ad* and *Ccp84Ad′* (colored circles in **Figure 7**). Other nonorthologous interactions can be identified from these dot plots (**Text S4**) that are also associated with these repetitive sequences. In *D. pseudoobscura*, there has been a gene conversion between *Ccp84Ab* and *Ccp84Ad* despite the fact that they are separated by *Ccp84Ac*. In *D. persimilis*, there has been an inversion of the region containing *Ccp84Aa* and *Ccp84Ab* the breakpoints of which coincide with the repetitive sequence. In contrast, there is no evidence of any non-orthologous interaction involving any of the genes in the 84A array that lack or have minimal repetitive sequence as visualized by dot plot, despite the fact that all genes in this array necessarily share substantial sequence similarity. The implications are that these repetitive sequences promote nonorthologous strand exchange, and that paralogous gene conversion occurs at a high rate in the germ line when nearby genes share this sequence. On the other hand, no nucleotide repeats were found to be associated with gene conversion in the 67F array.

**Figure 8:**
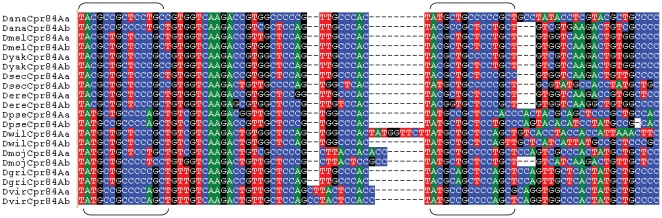
Alignment of *Ccp84Aa* and *Ccp84Ab* sequence identified as repetitive in dot plots. Nucleotide sequence was aligned with ClustalW and then trimmed around the most conserved repeat unit, although genes typically have more than two such units at varying degrees of conservation. Brackets indicate two copies of a repeated sequence that is well conserved among all species. Other repeats can be seen that are found in only a subset of species.

**Figure 8** shows an alignment of repetitive sequence from the *Ccp84Aa* and *Ccp84Ab* genes of each *Drosophila* each species. The repeats are not simple nucleotide repeats but are instead longer sequences that are inexactly repeated tens of nucleotides apart from each other. The repetitive sequence in this region has diverged among the different species but a core shared sequence can be identified (bracket in **Fig. 8**). This sequence is GC-rich and bears some similarity to GC-rich motifs associated with guanine ‘quadruplex’ pairing [33] and with recombination hotspots in human [34].

**Figure 9:**
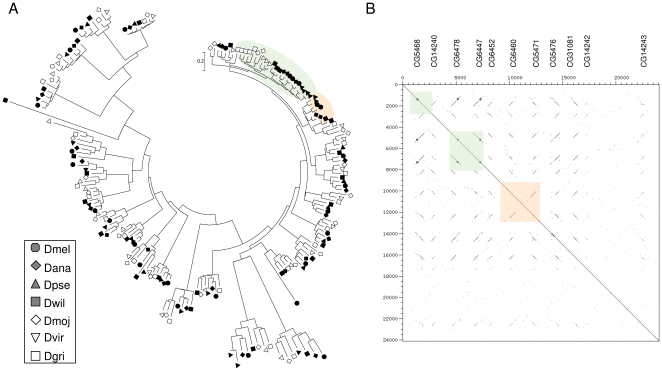
Concerted evolution of Drosophila Tweedle genes within an array at 97C. A. Neighbor-joining phylogeny of predicted proteins. B. Dot plot of array.

**Figure 10:**
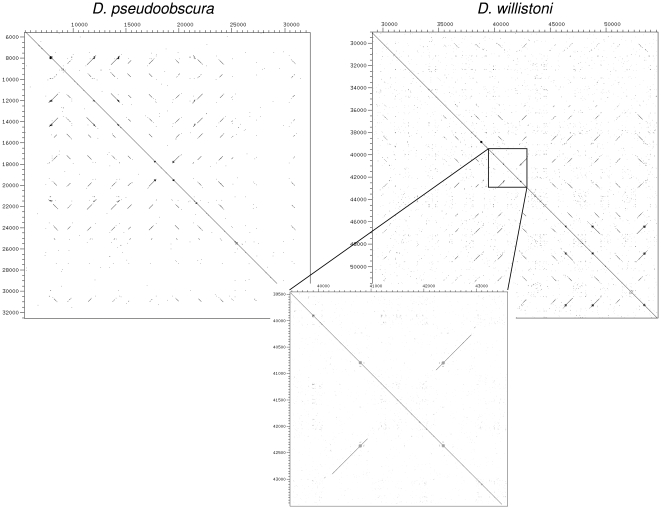
Dot plots of the co-orthologous regions of the *D. melanogaster* 97C Tweedle array from *D. pseudoobscura* and *D. willistoni*, with the region around *Dwil5471* and *Dwil6460* enlarged.

I then investigated whether within-species clustering of paralogs was associated with repetitive coding sequence in the Tweedle gene family. **Figure 9A** shows a phylogeny of all identified Tweedle genes in the seven *Drosophila* species. For most genes, putatively orthologous genes cluster together as expected under independent evolution, however, two sets of paralogous genes consistently cluster by species. Both sets of similar paralogs are within a larger array of Tweedle genes at 97C in *D. melanogaster*. *CG5468*, *CG6447*, and *CG6448* cluster as paralogs for all seven species (green shading) and *CG5471* and *CG6460* cluster within the Sophophora species group only (orange shading), as the latter gene is not present in the Drosophila species group. **Figure 9B** shows a dot plot of a tandem array of Tweedle genes in *D. melanogaster* that contains both groups. *CG5468*, *CG6447*, and *CG6448* each contain repetitive sequence near the 5′ end of the coding region whereas the other genes in the array do not. On the other hand, *CG5471* and *CG6460* have only a minimal amount of repetitive sequence, not visible by dot plot at this scale. These two genes also lack repetitive sequence in *D. ananassae* (not shown), but do have repetitive sequence in *D. pseudoobscura* and *D. willistoni* (**Figure 10**).

**Figure 11:**
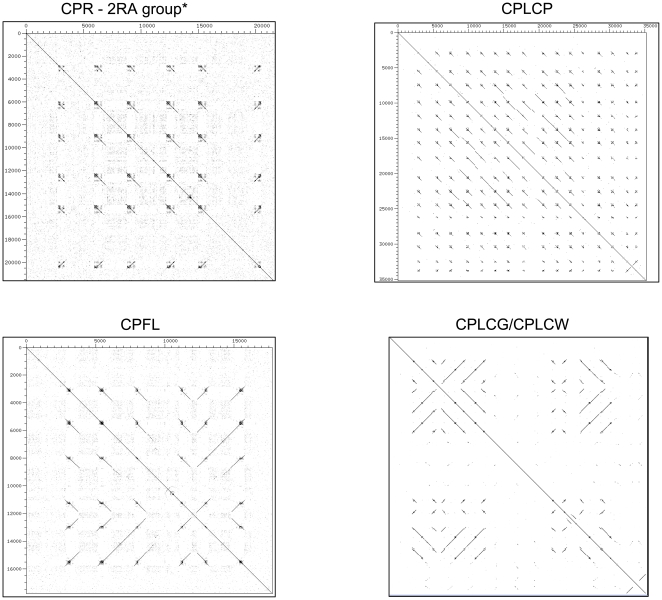
Dot plots of concertedly evolving cuticular protein genes of *An. gambiae*. (see [6], [7] for details). Additional dot plots are shown in Text S4.

Because *An. gambiae* cuticular proteins of the CPR and other multigene families show extensive concerted evolution [6], [7], I also used dot plots to look for similar patterns of nucleotide repetition associated with gene conversion tracts in this species. Comparable nucleotide repeats within coding regions were found for all sets of highly similar genes in *An. gambiae*, although the level of repetition varied substantially (examples are shown in **Fig. 11** and **Text S4**). In contrast, comparable repeats are largely absent for single-copy genes (not shown). Dot plots of some *An. gambiae* gene arrays also reveal extensive tandem duplication and/or inversions involving multiple genes. Thus, while the association between nucleotide repeats and concerted evolution is remarkably similar in both *Drosophila* and *An. gambiae*, specific pairs of interacting genes were not evident in the mosquito and the arrays are structurally more dynamic.

### Rates of Protein Evolution

The first part of this paper examined patterns of stasis and change in the number of genes within gene families. I then examined patterns of concerted evolution within tandem arrays of CPR genes. I now turn to investigating the evolutionary rate of change in the coding sequences themselves, which is usually quantified as the ratio of nonsynonymous substitutions (Ka) to synonymous substitutions (Ks). Estimating the Ka/Ks of orthologous sequences reveals how the same gene inherited from a common ancestor has diverged among the descendant species. Ka/Ks can be derived directly from Ka and Ks estimates for any pairwise comparison, but for sequences evolving along a phylogeny the ratio is parameterized as ω in PAML [29]. For a given phylogeny, the likelihoods of different evolutionary models can be compared using assigned or estimated ω parameters. This approach provides a statistical framework for evaluating explicit models of sequence evolution that are of biological interest.

I first estimated ω along the seven *Drosophila* species phylogeny for 116 ‘single-copy’ genes (those judged to have one-to-one orthologs in other species by sequence and synteny) from all four gene families. In addition to identifying specific genes likely to be under positive selection during the evolutionary history of *Drosophila*, this analysis can address whether, on average, different cuticular protein families evolve at similar or different rates. This is an interesting topic given that these gene families differ in age and in the complexity of their conserved domains and likely have distinct functions in cuticle. However, initial pairwise comparisons of Ka/Ks between orthologous sequences revealed a complication, which was that evolutionary rates within the Drosophila species group consistently differed from those of the Sophophora species group, regardless of cuticular protein family. I therefore estimated separate ω's for the two species groups. I also estimated a third ω for the branch connecting the Drosophila and Sophophora groups because pairwise comparisons indicated consistently high rates of evolution along this branch (i.e., three branch labels in PAML, with Model = 2 and Nsites = 0; see **Fig. 1**).

**Figure 12:**
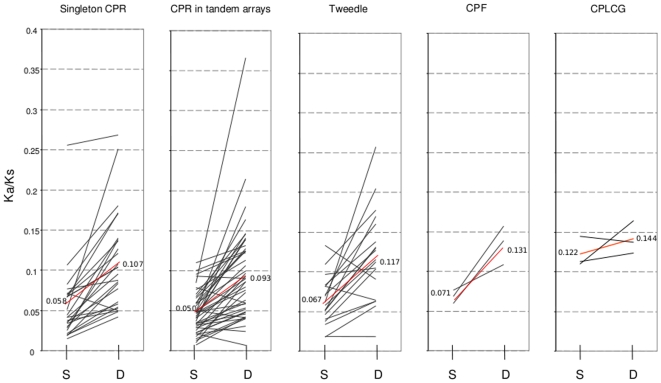
Graphs showing the range of ω estimates for orthologous ‘single-copy’ genes of each cuticular protein gene family, as described in the text. CPR genes occurring in tandem arrays or as isolated genes (‘singletons’) are shown separately. For each gene, the values in the Sophophora (S) and the Drosophila (D) species groups are shown separately, connected by a line to more clearly illustrate the trends among genes. The red lines indicate the mean values.

The distribution of ω estimates by gene family is shown in **Figure 12**. The range of evolutionary rates was similar for both singleton and tandemly arrayed CPR genes, and these rates were comparable to the Tweedle and CPF/CPFL families as well. The CPLCG family had the highest mean Ka/Ks, but the sample size is only three genes and the values are well within the range of the larger gene families. Thus, there does not appear to be a qualitative difference among these *Drosophila* cuticular protein families in rates of amino-acid evolution.

The results also confirmed that orthologous genes tend to have lower Ka/Ks within the Sophophora species group than within the Drosophila group (**Fig. 12**), implying higher rates of cuticular protein evolution in the latter group. However, this difference between species groups is primarily driven by shorter synonymous-site branch lengths within the Drosophila species group rather than longer nonsynonymous-site branch lengths (**Table 4**). That is, the total amount of amino-acid change has been similar within each group, but since synonymous substitution rates were lower in the Drosophila species group, the orthologous sequences in those species are therefore less conserved per unit of synonymous change. Codon bias is unlikely to explain this difference in Ks because the two species groups have very similar codon usage both for these genes (not shown) and, with the exception of *D. willistoni*, in general [35]. Furthermore, the proportion of indels in aligned sequences is also higher within the Sophophora group compared with the Drosophila group. **Table 5** shows the mean pairwise percentage of alignment sites with a gap, calculated separately for comparisons within Drosophila, within Sophophora, and between the two clades. For both the CPR and Tweedle families, the proportion of gaps was significantly higher in Sophophora by t-test (P<0.01 and P<0.05, respectively). These data suggest that demographic histories or mutation probabilities have differed between species groups.

**Table 4:** Mean evolutionary rates among orthologous single-copy genes in each species group, for all protein families combined.

Rate	Sophophora	Drosophila
Ka	0.035	0.041
Ks	0.676	0.383
ω*	0.056	0.103

* Note that the mean of ω estimates need not equal the ratio of mean Ka to mean Ks.

**Table 5:** Mean proportion of aligned sites containing gaps, within and between Drosophila and Sophophora species groups.

Gene family	Sophophora	Drosophila	Inter-group comparison
CPR**	0.077	0.036	0.079
Tweedle*	0.066	0.041	0.073

* P<0.05 of equal means by t-test.

** P<0.01 of equal means by t-test.

In contrast, PAML analysis strongly supported the initial observation of high evolutionary rates along the branch connecting the Drosophila and Sophophora species groups. Thirty-six out of the 116 single-copy genes examined had ω estimates along this branch equal to or greater than one, including all three CPF/CPFL genes. For those genes with an estimated ω less than one, the rate was nonetheless 3.3 times higher, on average, than the ω along other branches. Thus, the divergence of these two taxa was associated with a very broad acceleration in amino-acid substitutions within the cuticular proteome. This finding is unlikely to be an artifact of applying a complex model to a short and/or mutationally saturated branch of a gene tree, because estimates of κ were generally well below 2 and the synonymous branch lengths were generally moderate and consistent. Genes with unusually long synonymous branch lengths were discounted, as were cases of ambiguous orthology (such as concertedly evolving genes). Moreover, the topology of the species tree is very well supported by independent data [35].

To test whether any of these 36 genes had ω *significantly* greater than one, implying positive selection, requires a two-ω evolutionary model in PAML. In this model, one parameter represents the branch of interest and the other parameter represents the rest of the orthologous gene tree (i.e., Model = 2 and Nsites = 2). The likelihood of the gene trees is then estimated with ω fixed at 1 or ω free to vary, and the two likelihoods compared by a χ^2^ test [36]. Using this test, I identified 13 genes with Ka/Ks statistically greater than 1 during the divergence of the two species groups (**Table 6**). The CPR genes identified tend to be highly diverged from paralogs as well, i.e they lie on long branches in the *Drosophila* CPR phylogeny [6], [13], and their products include the eye lens protein drosocrystallin [37], [38] and the *Drosophila* resilin protein [39].

**Table 6:** Genes with statistically significant evidence of positive selection along branch connecting the Sophophora and Drosophila species groups.

Family	*D. melanogaster* gene name	ln(likelihood), ω fixed = 1	ln(likelihood), estimated ω>1	2Δln(likelihood)*
CPR	*resilin*	−5503.0	−5487.0	31.89
Tweedle	*TweedleX*	−3593.5	−3581.5	23.88
CPR	*Cpr100A*	−2010.7	−2001.8	17.73
CPR	*Cpr76Bd*	−15292.6	−15284.1	16.99
CPR	*Cpr47Ee*	−3945.6	−3939.3	12.44
CPR	*Cpr65Ec*	−1336.6	−1330.7	11.79
Tweedle	*TweedleV*	−1936.6	−1930.8	11.59
CPR	*Cpr72Ea*	−2067.2	−2061.7	10.99
CPR	*cry (crystallin)*	−4135.8	−4130.6	10.44
CPR	*Cpr30B*	−1654.3	−1649.9	8.83
CPR	*Cpr66Cb*	−1392.0	−1389.3	5.31
CPR	*Cpr72Ec*	−3220.8	−3218.7	4.08
CPR	*Cpr49Af*	−1697.7	−1695.7	4.08

* Critical value of χ^2^ distribution is 3.84 for α = 0.05 and 1 degree of freedom.

## Discussion

There are scores of proteins that contribute to cuticle in *Drosophila*, about which relatively little is known. The goal of this study was to characterize evolutionary patterns within the *Drosophila* cuticular proteome, in order to gain insight into how this protein diversity has evolved, both within the genus and compared with other Diptera. The results show that the rate of change in the number of cuticular protein genes is much slower (in absolute time) within *Drosophila* than along the branches connecting *Drosophila* with the mosquitoes *An. gambiae* and *Ae. aegypti*. Thus, the differences in gene number between genera do not merely reflect greater divergence times, but instead suggest periods of more rapid evolutionary change and subsequent stasis. For comparison, the rate of change in chemosensory gene number is as high or higher within *Drosophila* as it is among *Drosophila* and *Anopheles*, based on CAFE results for data reported by [40]. In that case, the most likely value of λ was 7.0E-3 within *Drosophila* and greater than or equal to 3.8E-3 among *Drosophila* and *Anopheles* (again, higher values for the latter group could not be evaluated as noted previously). Thus, these two categories of gene family appear to have had different rates of evolution during the diversification of Drosophila. It will be of great interest to determine whether differences in cuticular protein complement are associated with the greater morphological complexity of mosquito larvae and pupae relative to *Drosophila*.

An important feature of gene-family evolution highlighted in this study is that, in *Drosophila*, gene turnover and/or gene conversion are primarily concentrated in a few tandem arrays of CPR genes. The functions of these gene products during cuticle assembly may dictate their susceptibility to duplication or loss. Future comparative analyses of the regulation and function of cuticular proteins, expanding on work already begun on the 65A array [5], [31], [41], [42], [43], might help explain this evolutionary pattern. Alternatively, local sequence and/or chromatin features, such as intergenic repetitive elements, could contribute to variation in rates of structural mutation. Such factors seem unlikely in the case of the 65A array, in which well-conserved genes and highly labile genes are interspersed together, but may play a role in the evolution of the 44C array.

Nucleotide sequence repetition within coding regions was associated with recurring gene conversion between particular genes within the 84A array of CPR genes and within the 97C array of Tweedle genes. These patterns led me to extend this correlation between coding sequence repeats and concerted evolution to sets of highly similar cuticular protein genes in *An. gambiae* as well. While the exact nature and extent of sequence repetition clearly varies among sets of concertedly evolving genes (**Text S4**), this strong and consistent association in both *Drosophila* and *Anopheles* suggests a common molecular mechanism, one that may be present in other Diptera. Given the importance of this order in agricultural systems and for human health, there will doubtless be additional genomic data in the near future with which to evaluate this hypothesis. In addition to these genomic data, population-level and chromatin-level studies of this phenomenon are also needed to understand the process of gene conversion at these sites. However, the possibility that the mechanism is related to known associations between GC-rich sequence and both quadraplex pairing [33] and recombination [34] seems promising.

The adaptive significance of concerted evolution of cuticular proteins remains to be clarified. Gene conversion events may be selectively neutral or may be an example of gene amplification in response to some selective pressure, such as the rate of protein production. A number of examples of adaptive gene amplification have been identified, primarily in cases of microbial adaptation to nutrient limitation and in human cancers [44]. A more relevant example is the developmentally programmed amplification of chorion genes within follicle cells during *Drosophila* oogenesis [45]. The rapid synthesis of specific proteins at discrete points during development characterize both chorion and cuticle synthesis and it is reasonable to hypothesize that concerted evolution with the *Drosophila* CPR family is adaptive for this purpose. In this regard, it is noteworthy that conversion tracts occasionally extend both upstream and downstream of genes, yet only the portion of the second exon containing the (chitin-binding) R&R Consensus is consistently homogenized within each species (see **Fig. 4B**, **Fig. 5B** and **Fig. 6**). This suggests that there is no intrinsic bias in the direction that strand exchange proceeds, but rather that mutations that homogenize this portion of the gene are more likely to be fixed. Whether genes undergoing gene conversion are highly expressed at the per-cell level remains to be clarified, although products of the 44C array are known to be abundant in third instar larvae [46].

Other examples of interlocus gene conversion reported in insects include the homogenization of heat shock protein genes in the mosquito *An. albimanus* [47], [48] and in *Drosophila* [49]. These proteins are also likely to be highly expressed at certain times and, given their role in protein folding, might be reasonable candidates for selection for homogeneity. Dot plots of heat shock genes (not shown) did not reveal any repetitive sequence comparable to that observed in this study. These examples, along with the concerted evolution of *Cpr67Fa1* and *Cpr67Fa2* described here, further demonstrate that nucleotide repeats are not required for concerted evolution. Rather, a diversity of factors is likely to influence the underlying rates and fixation probabilities of conversion events among different genes. More extensive population-genetic data from a set of more closely related *Drosophila* species would help confirm these results and potentially identify sites under selection.

This study identified a consistent increase in the rate of cuticular protein sequence evolution along the branch connecting the Sophophora and Drosophila species groups. Indeed, a number of proteins had Ka/Ks significantly greater than one, implying positive diversifying selection, perhaps in response to new developmental or ecological conditions. This finding demonstrates that cuticular protein evolution at the amino-acid level is a potentially important component of ongoing adaptation of insect species. The genes identified here as having evolved under positive selection are favorable candidates for functional/ecological studies of species divergence.

Numerous genome-scale analyses have sought to infer general patterns in the evolution of duplicated genes, such as changes in ploidy or gene number, rates of nonsynonymous substitution, and divergence in gene expression. While these studies have provided extraordinary insights, focusing on the evolution of individual families can more directly connect the molecular histories of duplicated genes to their biological functions and effects on fitness. Insect cuticle promises to be an increasingly powerful model for studying, at hierarchical scales, the origins of biological novelty.

## Supporting Information

Text S1 Sequences of genes/proteins used in this study. *Drosophila melanogaster* names are those used by Ensembl, whereas predicted sequences from other *Drosophila* species have names combining a four-letter species code and an arbitrary number. File is a spreadsheet with separate pages for each family of cuticular protein genes examined in the text. Sequences are entered in fasta format and can be copied or saved as text.
(0.36 MB XLS)

Text S2 Tables showing inferred orthologous genes, or inferred co-orthologous groups of genes when one-to-one matching is not possible.
(0.04 MB XLS)

Text S3 Phylogenies of CPR genes from seven *Drosophila* species, grouped by tandem array. Each slide shows a phylogenetic tree representing tandemly arrayed CPR genes from seven *Drosophila* species as indicated by the legends. A tree is shown for each tandem array listed in Table 3, except for those at chromosomal band 44C and 65A of *D. melanogaster*, which are shown in Figure 2 and Figure 3, respectively. All trees are neighbor-joining trees constructed from predicted protein sequences using the JTT cost-exchange matrix, as described in the methods. There is no methodological difference between trees presented with circular versus rectangular branches. Rather, the former is used when the number of genes is too large to be easily presented in rectangular format.
(1.76 MB PPT)

Text S4 Dot plots of tandem arrays of CPR genes in *Drosophila* species and in *Anopheles gambiae*. The first slide shows dot plots of the 84A region in ten *Drosophila* species, to show the generality of the pattern shown in Figure 3 of the manuscript. The remaining slides show patterns of repetitive sequence within tandem arrays of CPR genes in *Anopheles gambiae* (see cited references in text).
(2.67 MB PPT)
