# Supplementary material for: Molecular Evolution of Drosophila Cuticular Protein Genes
Source: PLoS One. 2009 Dec 17;4(12):e8345. doi: 10.1371/journal.pone.0008345 (PMC2793513; doi:10.1371/journal.pone.0008345)
Supplement: Text S3 — Phylogenies of CPR genes from seven Drosophila species, grouped by tandem array. Each slide shows a phylogenetic tree representing tandemly arrayed CPR genes from seven Drosophila species as indicated by the legends. A tree is shown for each tandem array listed in Table 3, except for those at chromosomal band 44C and 65A of D. melanogaster, which are shown in Figure 2 and Figure 3, respectively. All trees are neighbor-joining trees constructed from predicted protein sequences using the JTT cost-exchange matrix, as described in the methods. There is no methodological difference between trees presented with circular versus rectangular branches. Rather, the former is used when the number of genes is too large to be easily presented in rectangular format. (1.76 MB PPT) [file pone.0008345.s003.ppt]

## Slide 1
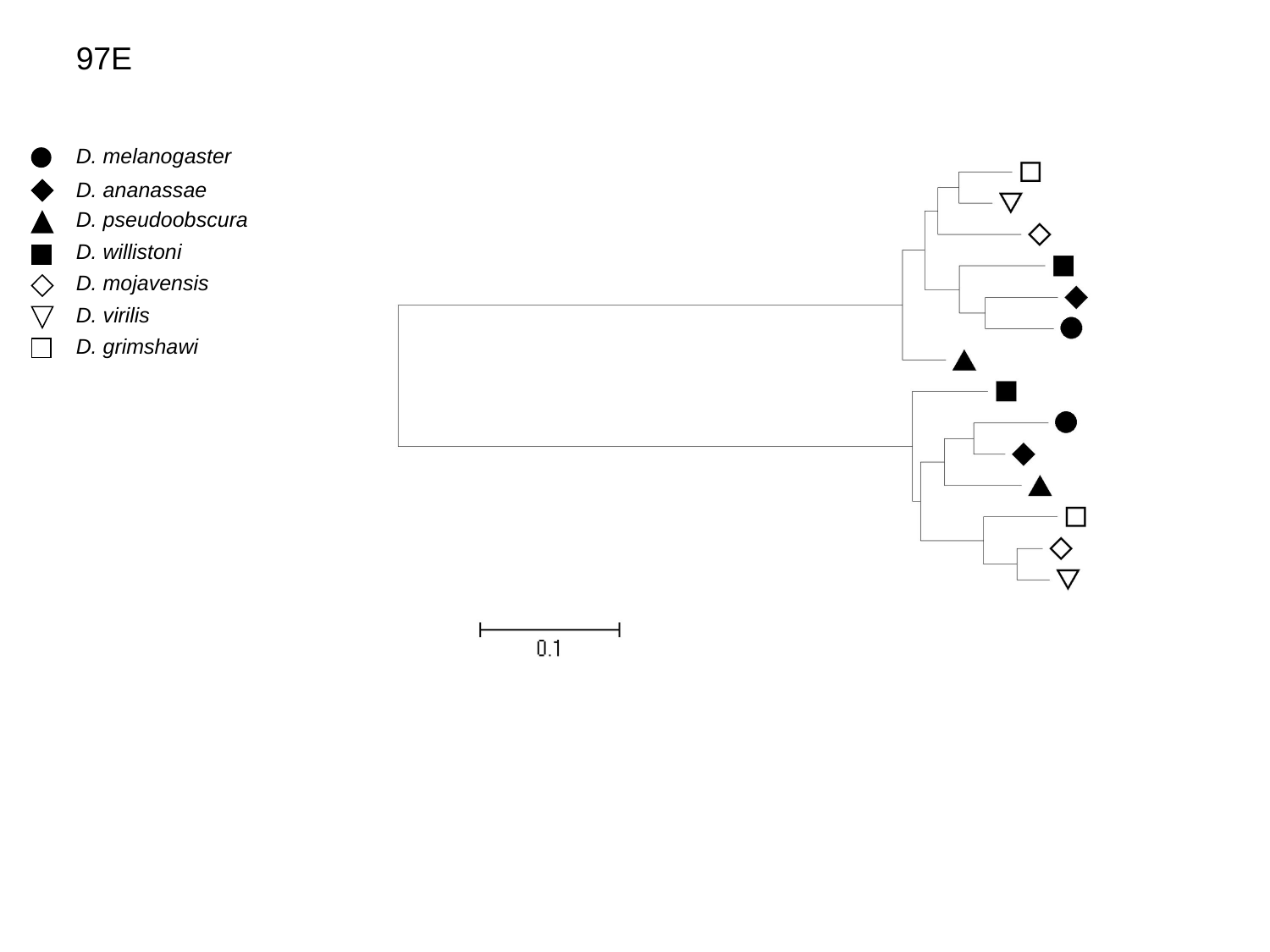

97E
D. melanogaster
D. ananassae
D. pseudoobscura
D. willistoni
D. mojavensis
D. virilis
D. grimshawi

## Slide 2
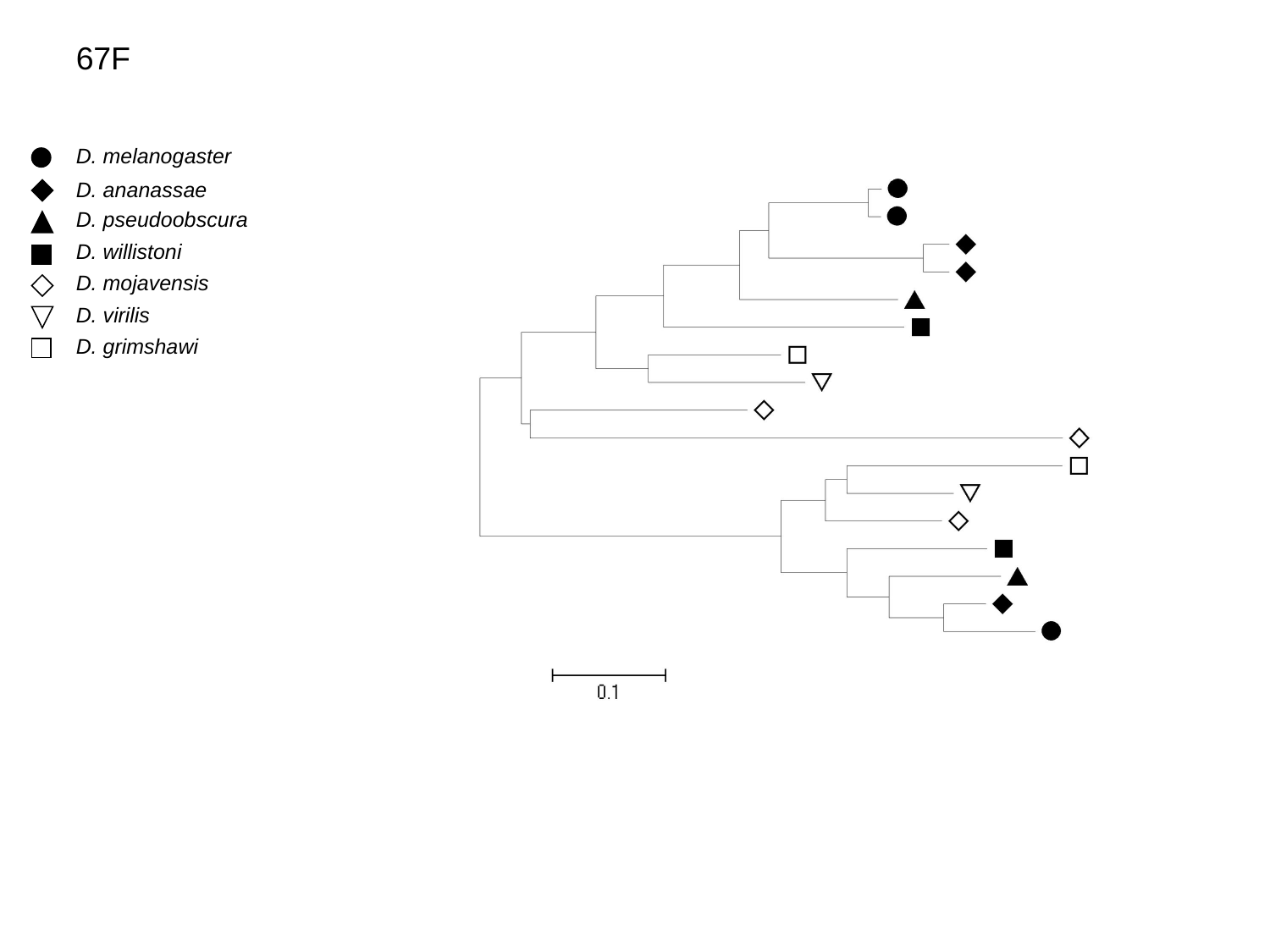

67F
D. melanogaster
D. ananassae
D. pseudoobscura
D. willistoni
D. mojavensis
D. virilis
D. grimshawi

## Slide 3
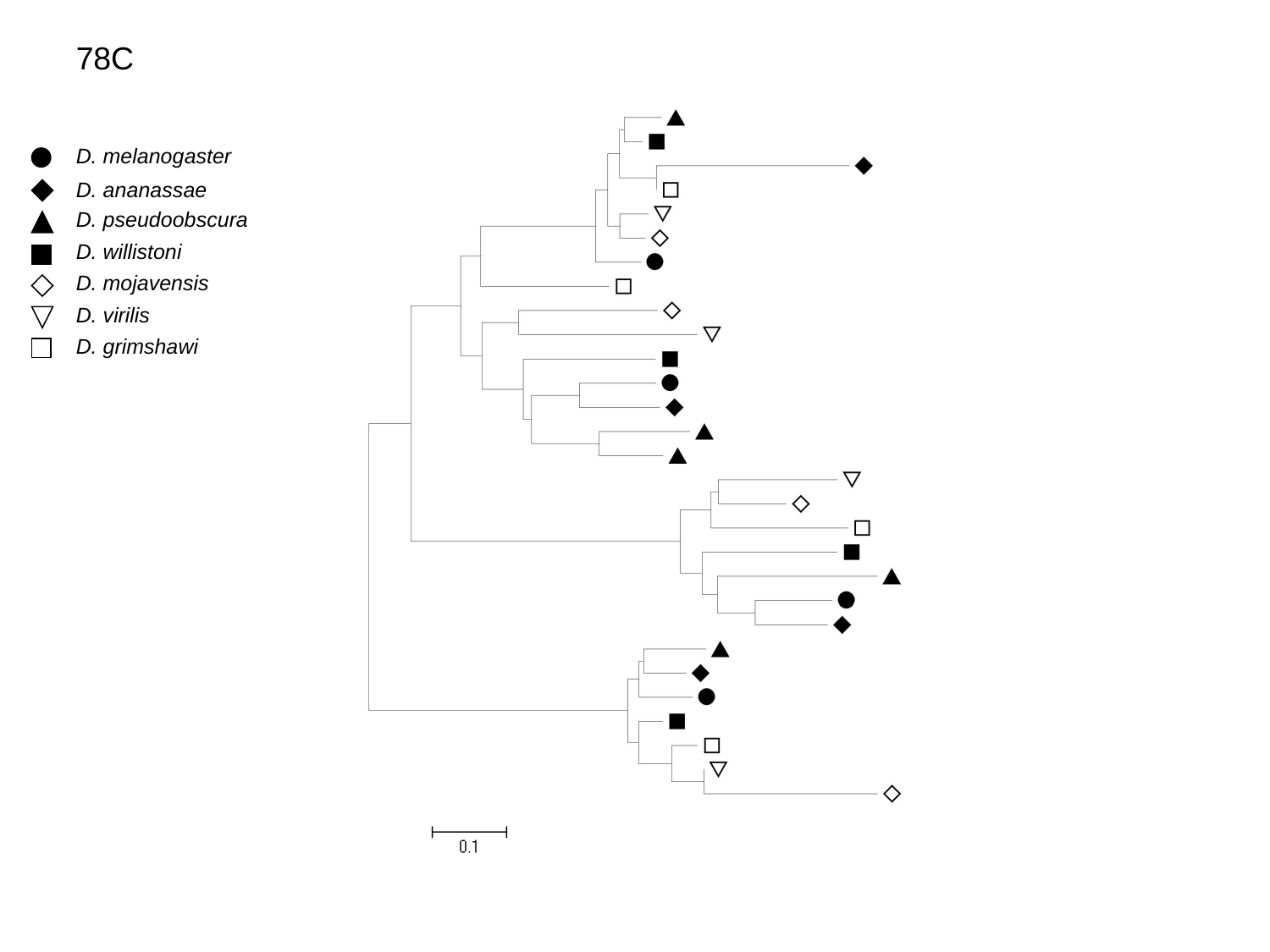

78C
D. melanogaster
D. ananassae
D. pseudoobscura
D. willistoni
D. mojavensis
D. virilis
D. grimshawi

## Slide 4
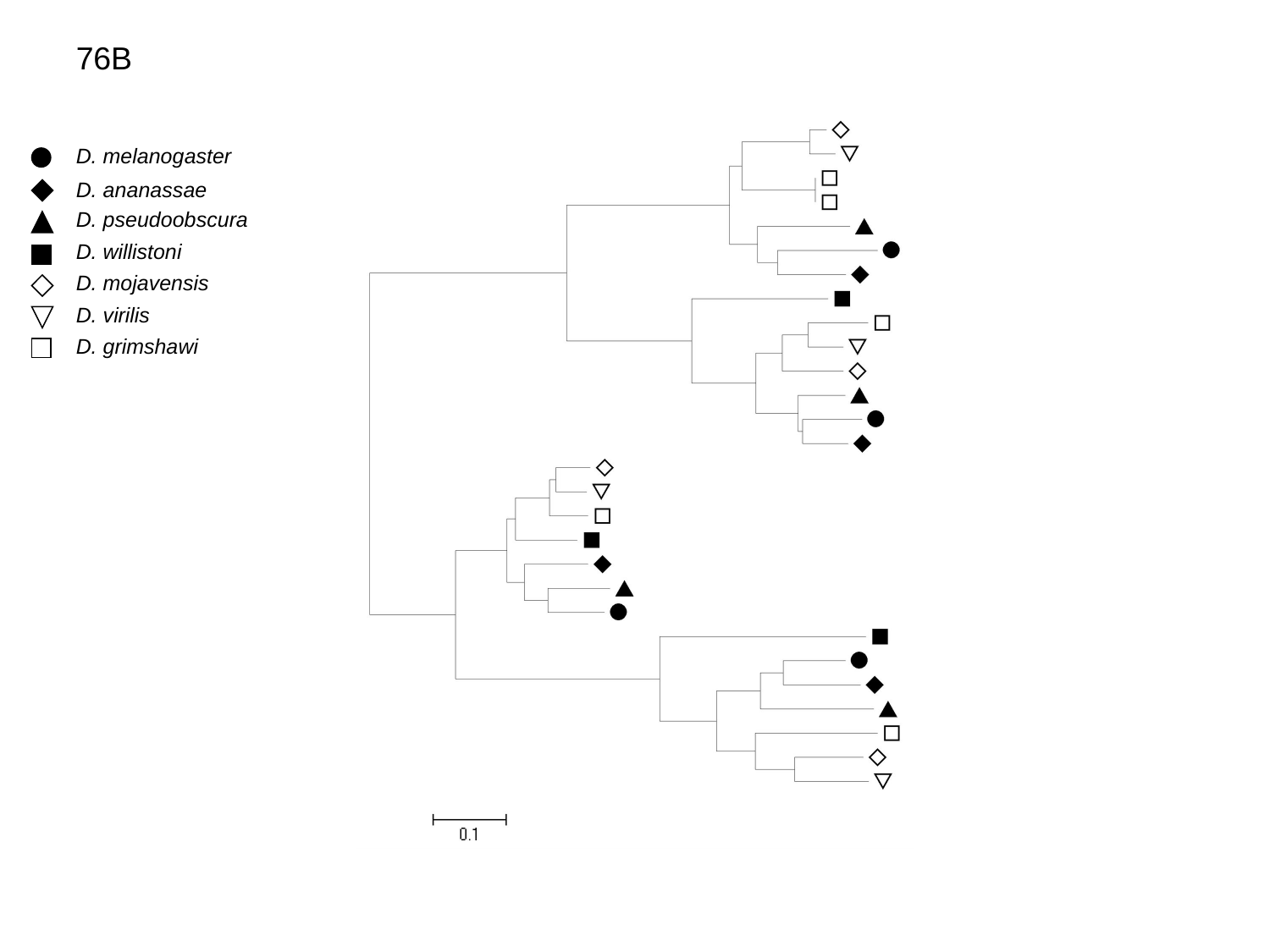

76B
D. melanogaster
D. ananassae
D. pseudoobscura
D. willistoni
D. mojavensis
D. virilis
D. grimshawi

## Slide 5
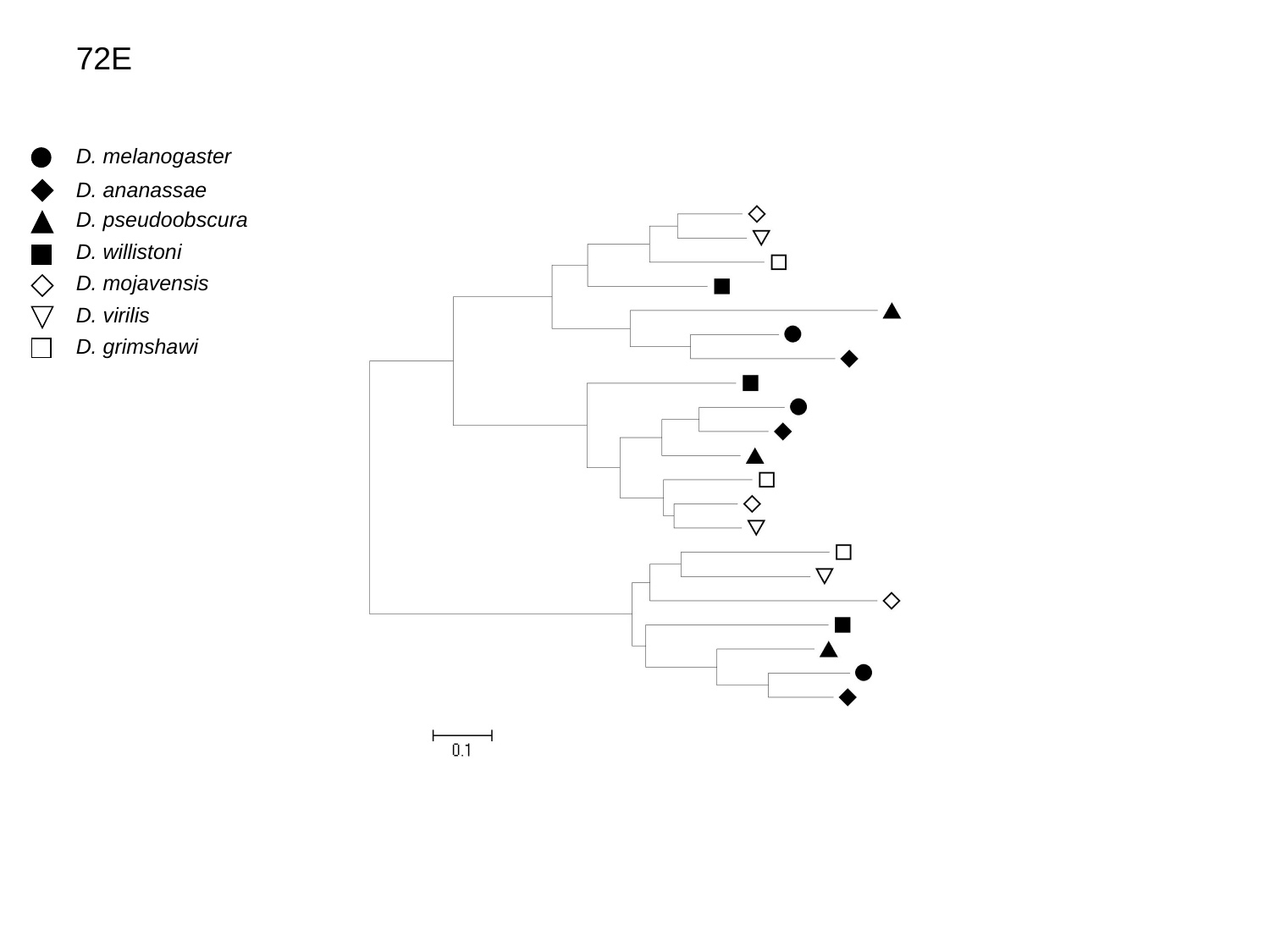

72E
D. melanogaster
D. ananassae
D. pseudoobscura
D. willistoni
D. mojavensis
D. virilis
D. grimshawi

## Slide 6
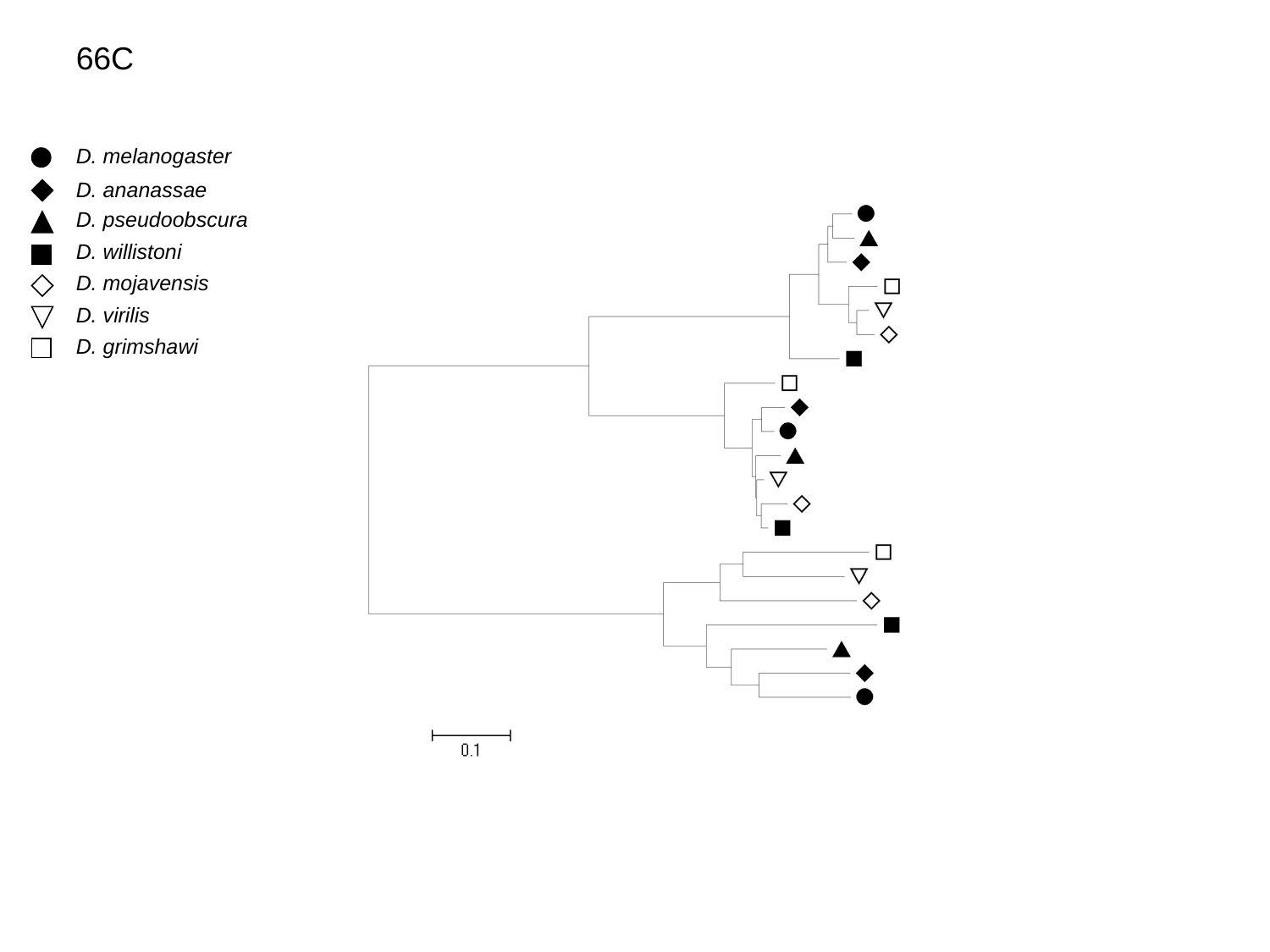

66C
D. melanogaster
D. ananassae
D. pseudoobscura
D. willistoni
D. mojavensis
D. virilis
D. grimshawi

## Slide 7
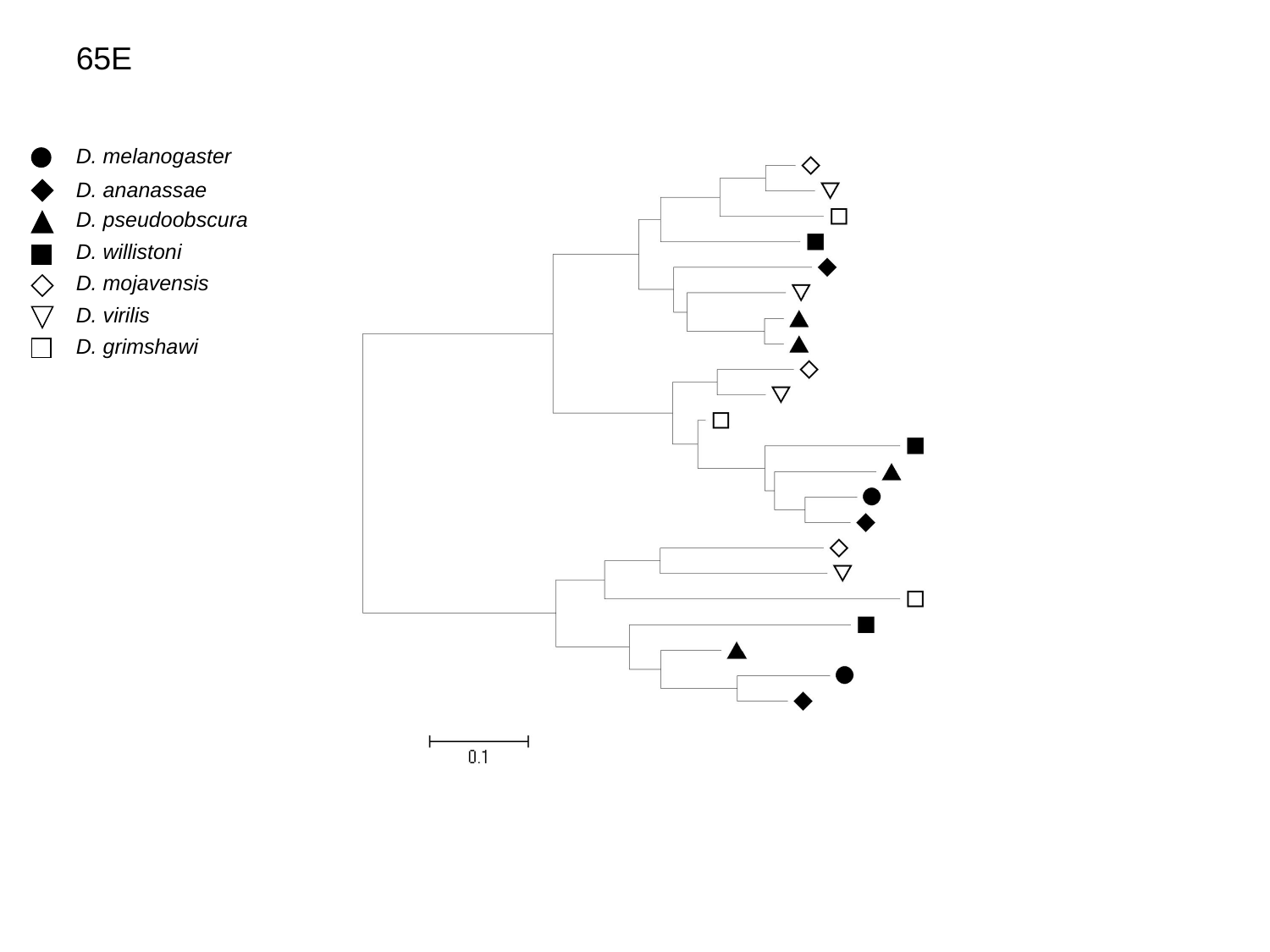

65E
D. melanogaster
D. ananassae
D. pseudoobscura
D. willistoni
D. mojavensis
D. virilis
D. grimshawi

## Slide 8
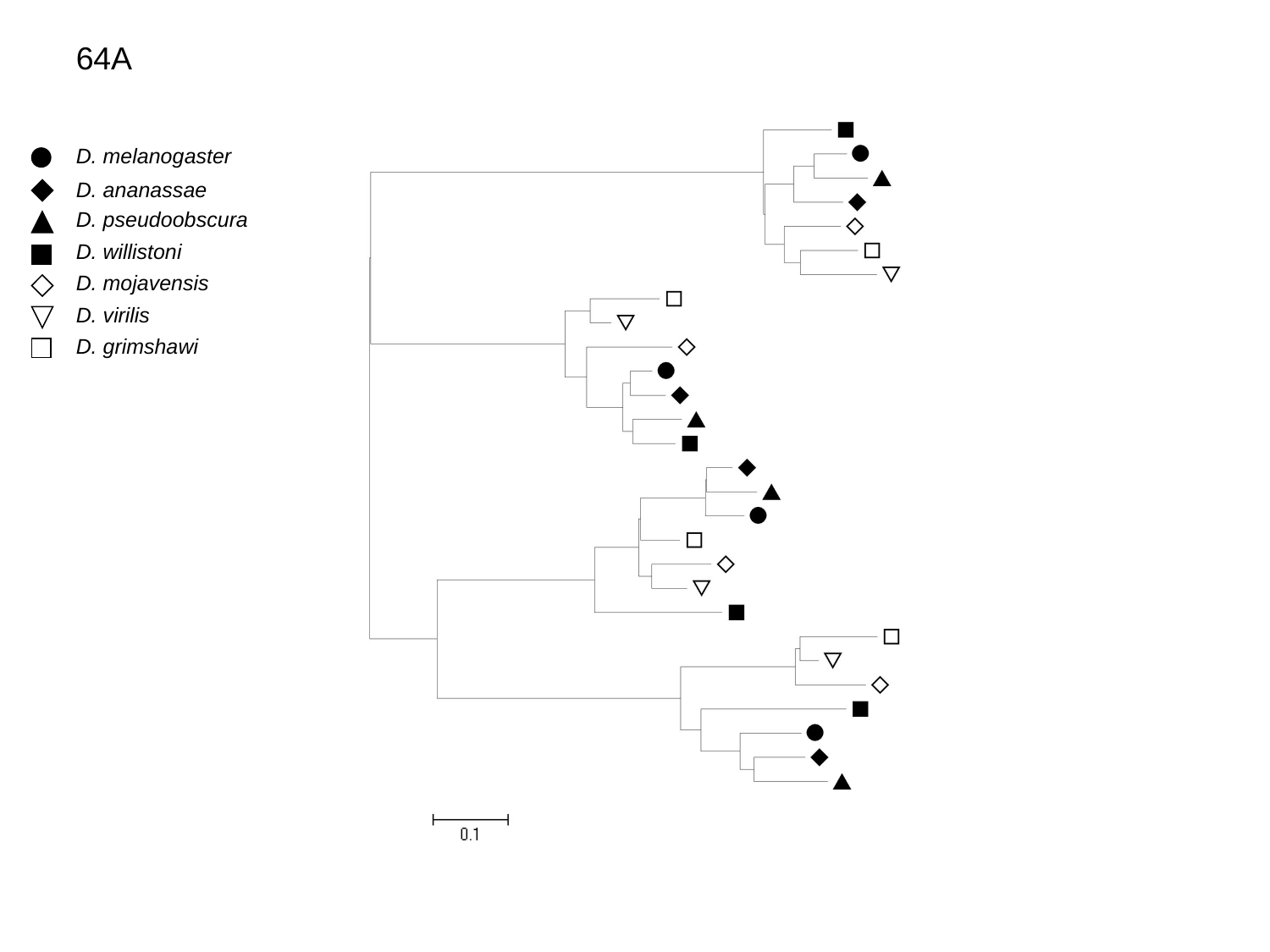

64A
D. melanogaster
D. ananassae
D. pseudoobscura
D. willistoni
D. mojavensis
D. virilis
D. grimshawi

## Slide 9
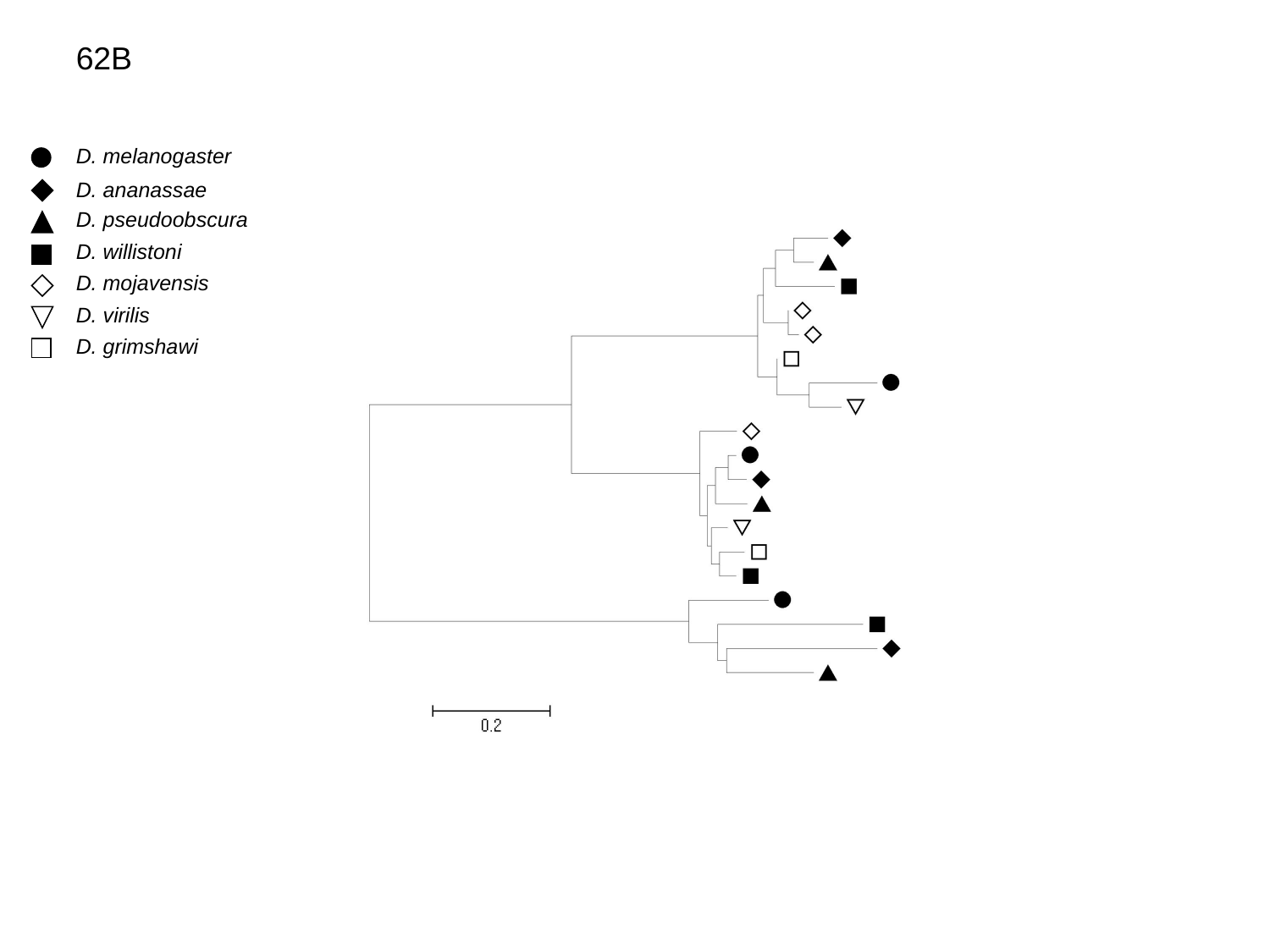

62B
D. melanogaster
D. ananassae
D. pseudoobscura
D. willistoni
D. mojavensis
D. virilis
D. grimshawi

## Slide 10
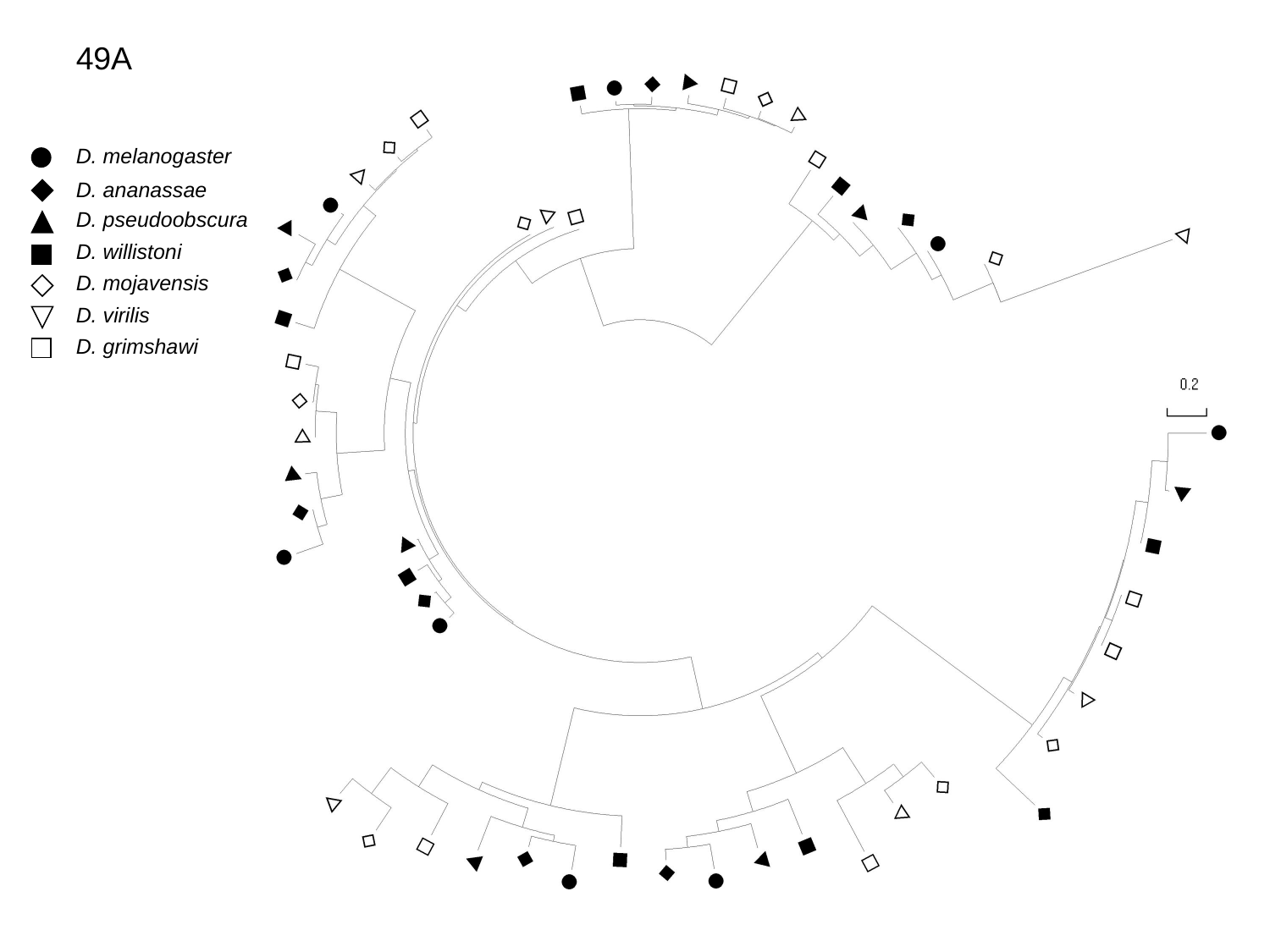

49A
D. melanogaster
D. ananassae
D. pseudoobscura
D. willistoni
D. mojavensis
D. virilis
D. grimshawi

## Slide 11
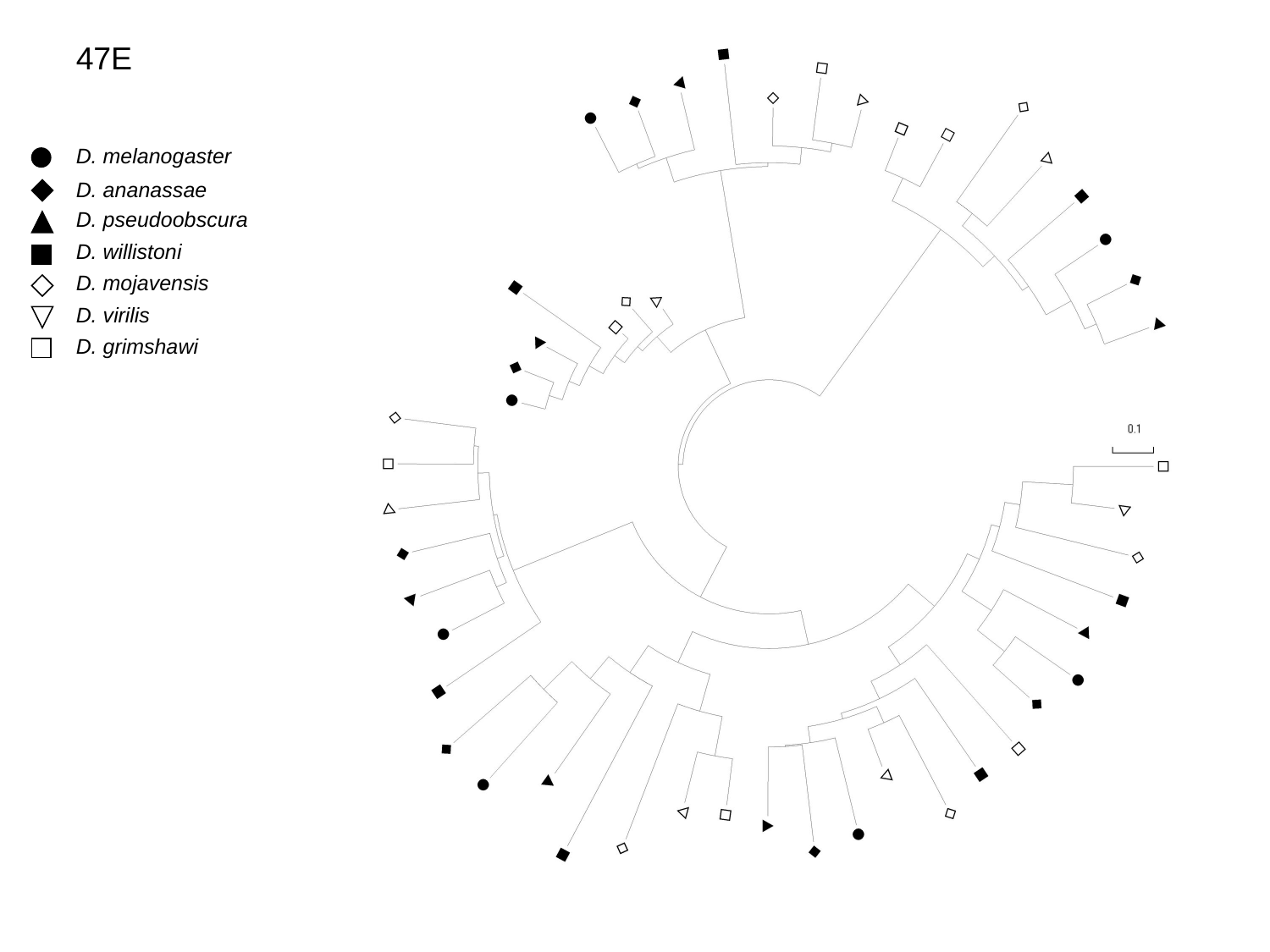

47E
D. melanogaster
D. ananassae
D. pseudoobscura
D. willistoni
D. mojavensis
D. virilis
D. grimshawi

## Slide 12
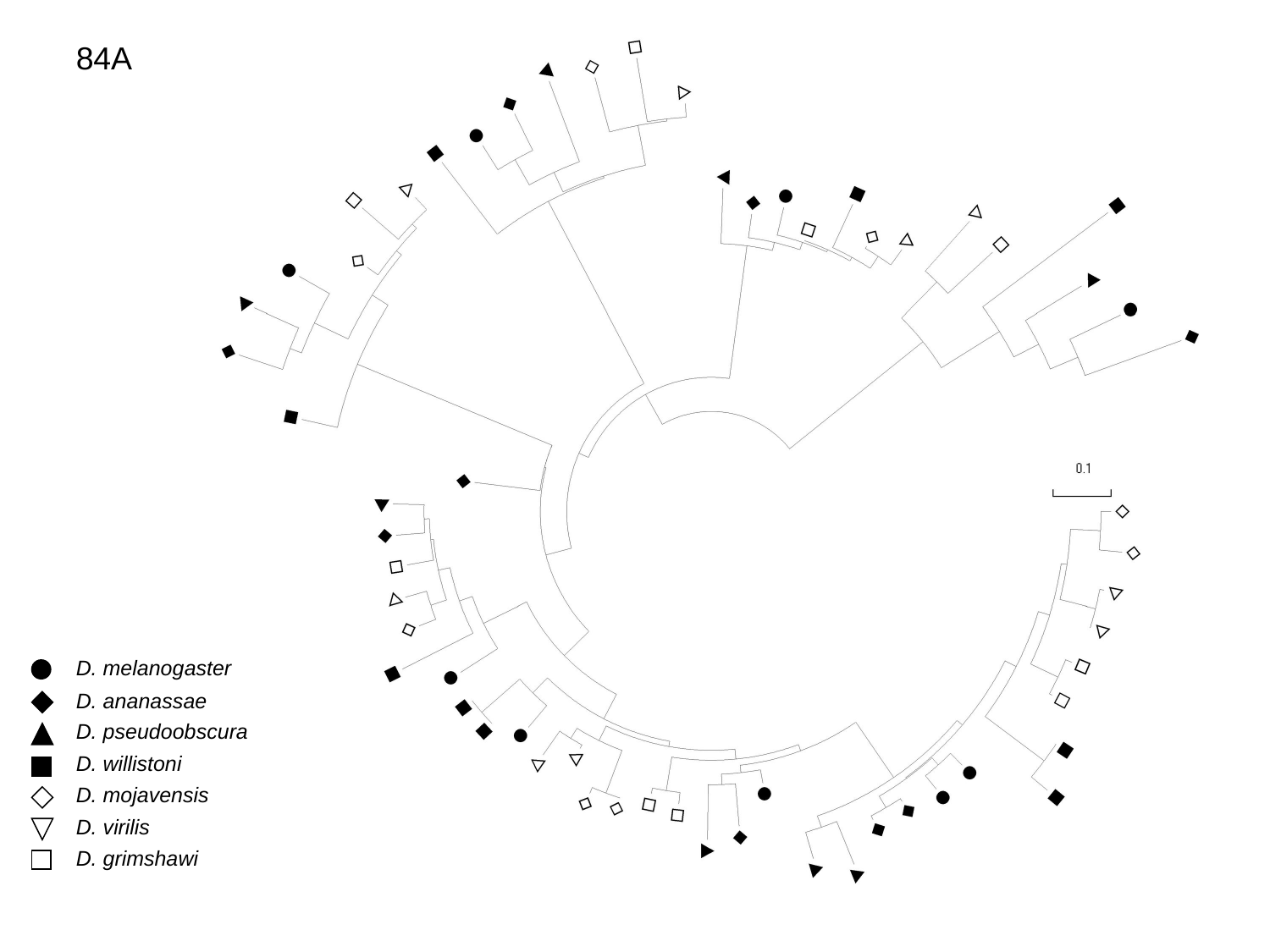

84A
D. melanogaster
D. ananassae
D. pseudoobscura
D. willistoni
D. mojavensis
D. virilis
D. grimshawi

## Slide 13
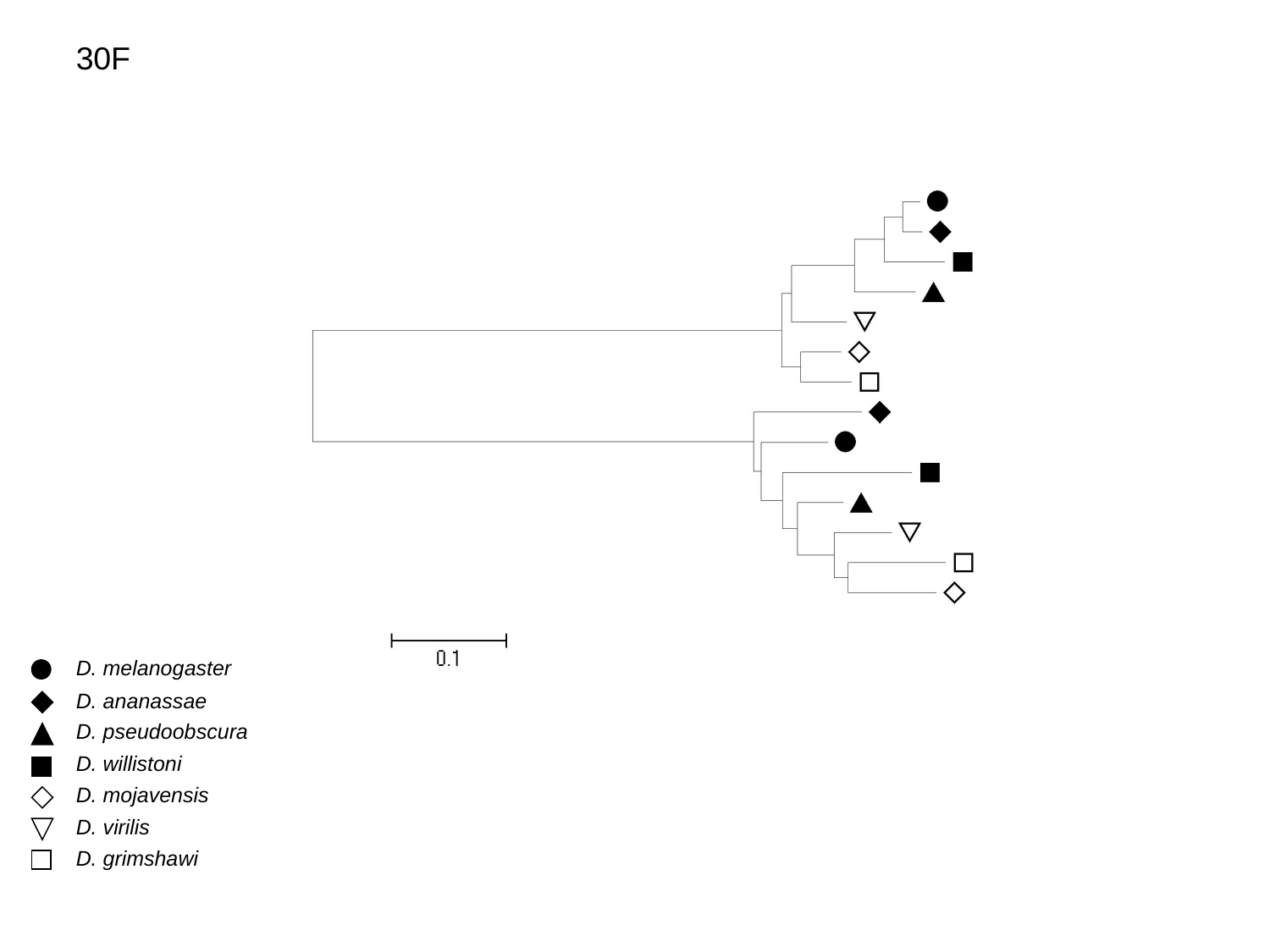

30F
D. melanogaster
D. ananassae
D. pseudoobscura
D. willistoni
D. mojavensis
D. virilis
D. grimshawi
