# Supplementary material for: Molecular Evolution of Drosophila Cuticular Protein Genes
Source: PLoS One. 2009 Dec 17;4(12):e8345. doi: 10.1371/journal.pone.0008345 (PMC2793513; doi:10.1371/journal.pone.0008345)
Supplement: Text S4 — Dot plots of tandem arrays of CPR genes in Drosophila species and in Anopheles gambiae. The first slide shows dot plots of the 84A region in ten Drosophila species, to show the generality of the pattern shown in Figure 3 of the manuscript. The remaining slides show patterns of repetitive sequence within tandem arrays of CPR genes in Anopheles gambiae (see cited references in text). (2.67 MB PPT) [file pone.0008345.s004.ppt]

## Slide 1
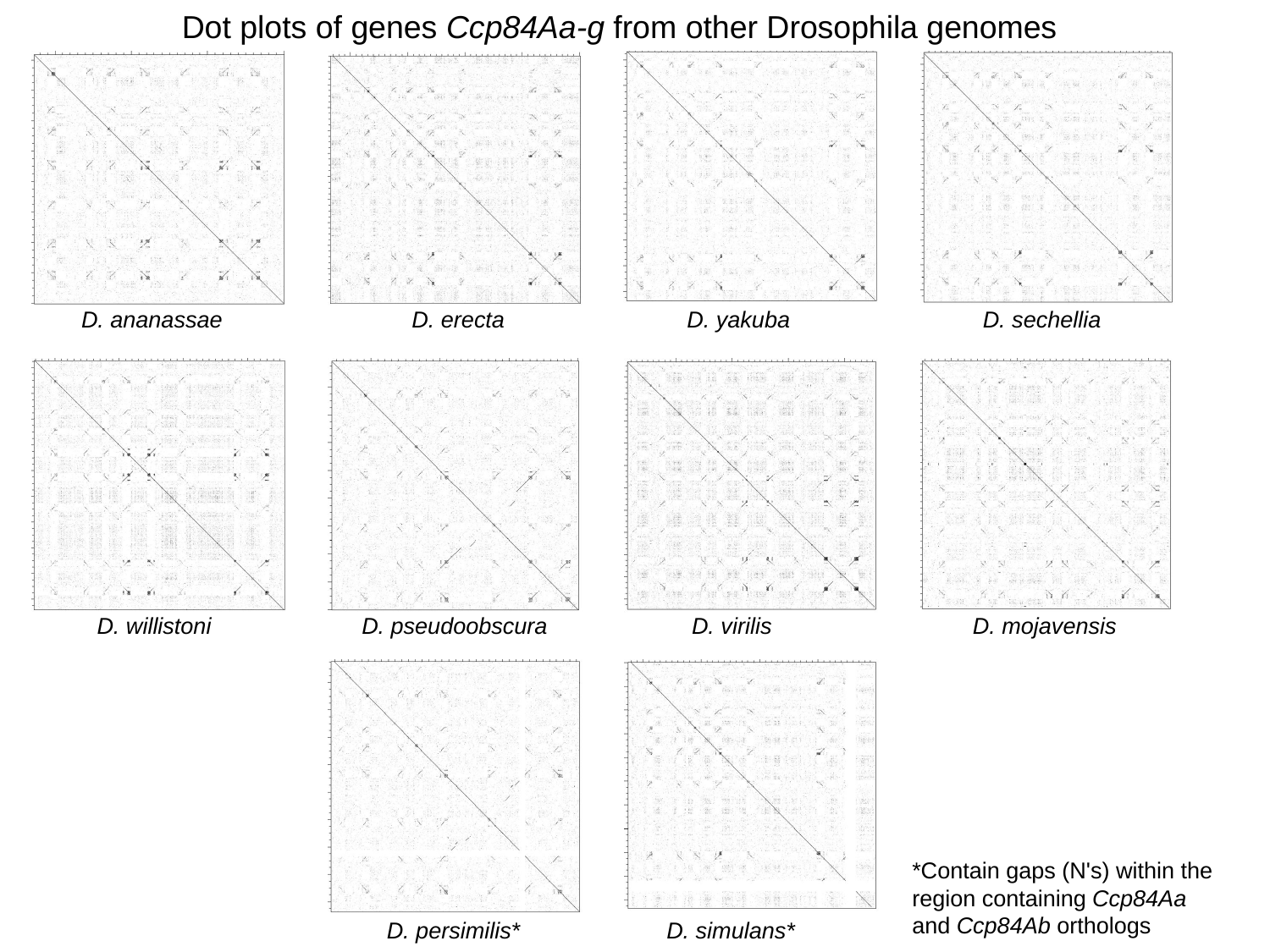

Dot plots of genes Ccp84Aa-g from other Drosophila genomes
D. ananassae
D. erecta
D. yakuba
D. sechellia
D. willistoni
D. pseudoobscura
D. virilis
D. mojavensis
*Contain gaps (N's) within the region containing Ccp84Aa and Ccp84Ab orthologs
D. persimilis*
D. simulans*

## Slide 2
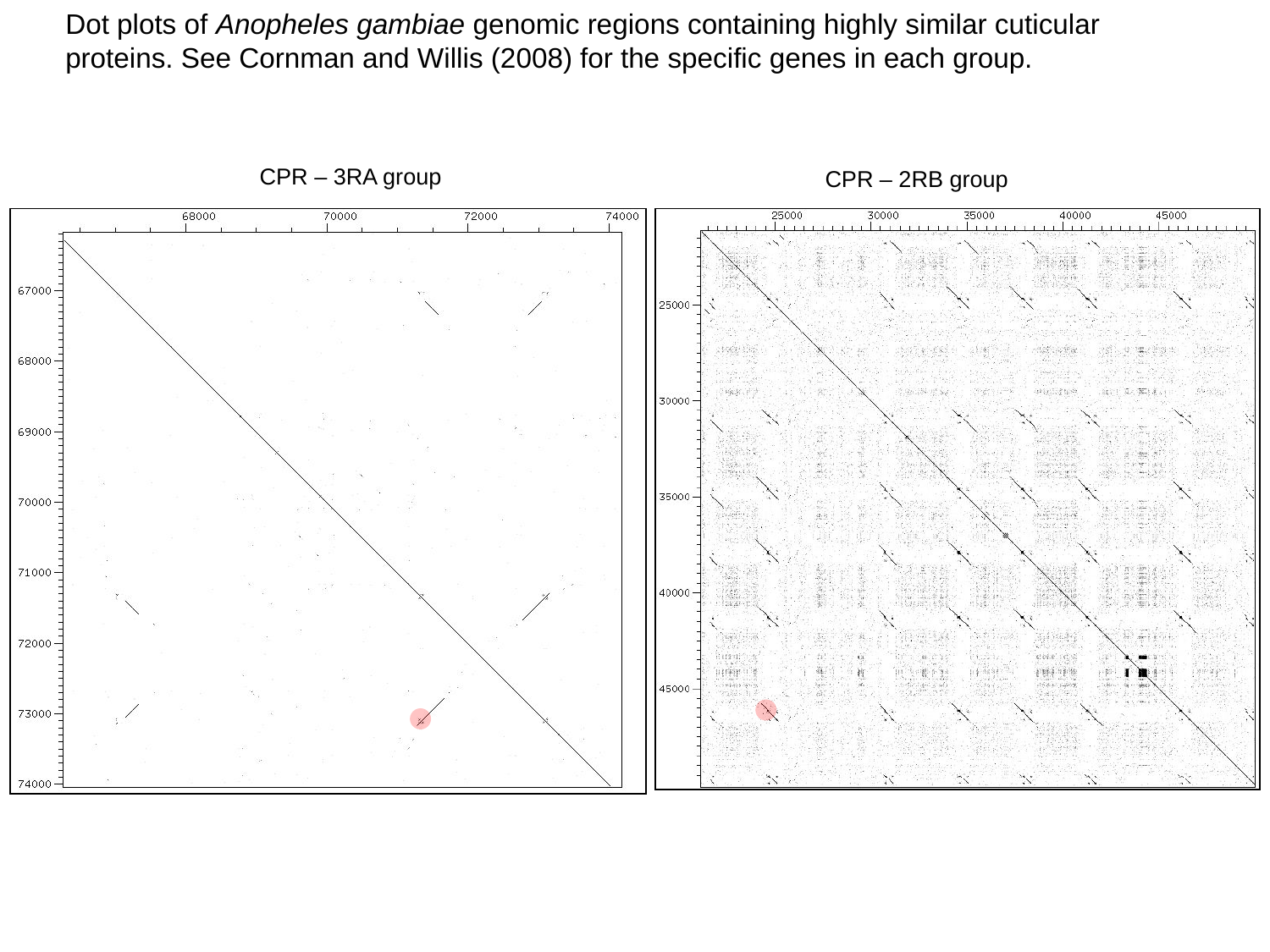

Dot plots of Anopheles gambiae genomic regions containing highly similar cuticular proteins. See Cornman and Willis (2008) for the specific genes in each group.
CPR – 3RA group
CPR – 2RB group

## Slide 3
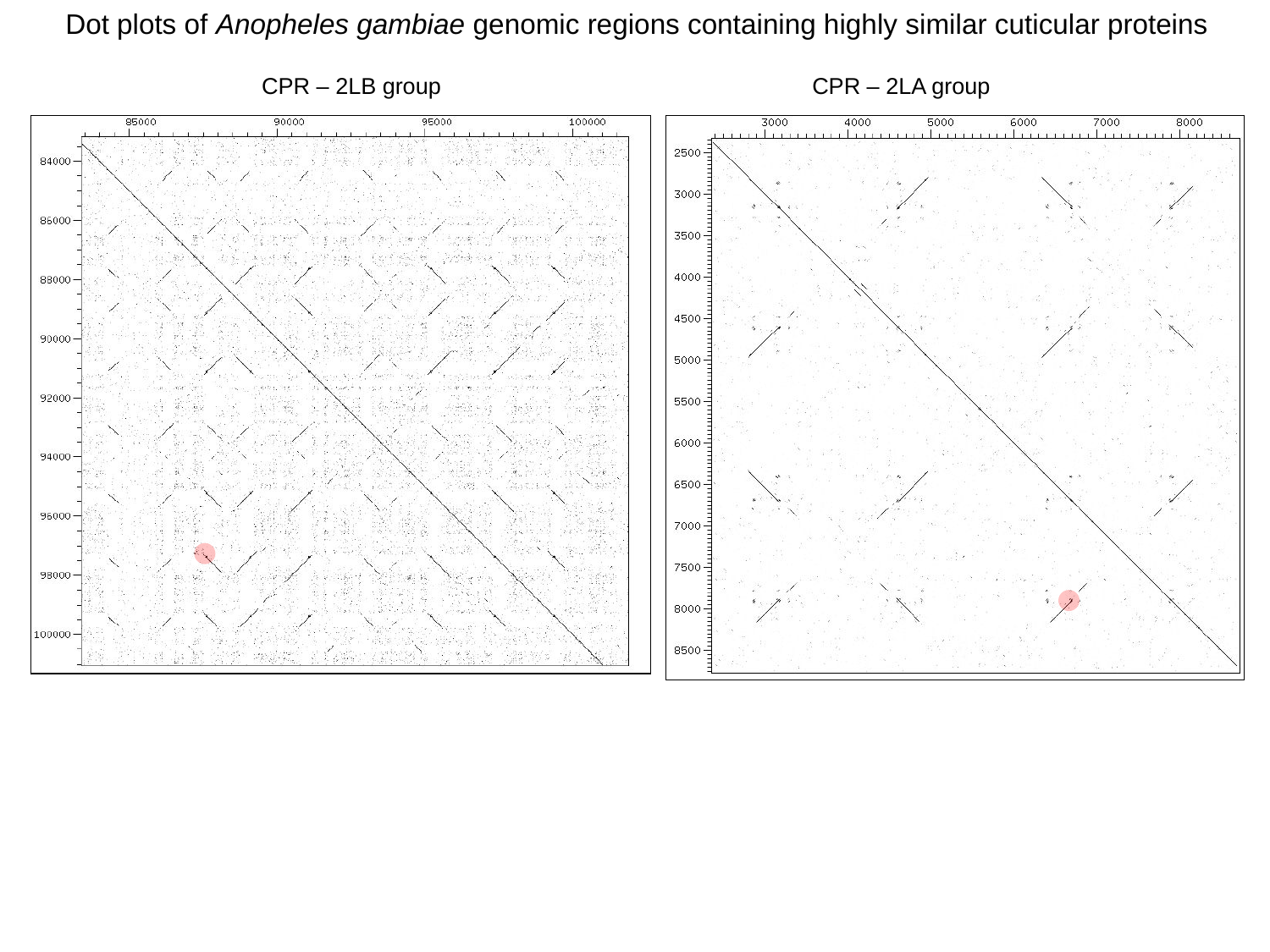

Dot plots of Anopheles gambiae genomic regions containing highly similar cuticular proteins
CPR – 2LB group
CPR – 2LA group

## Slide 4
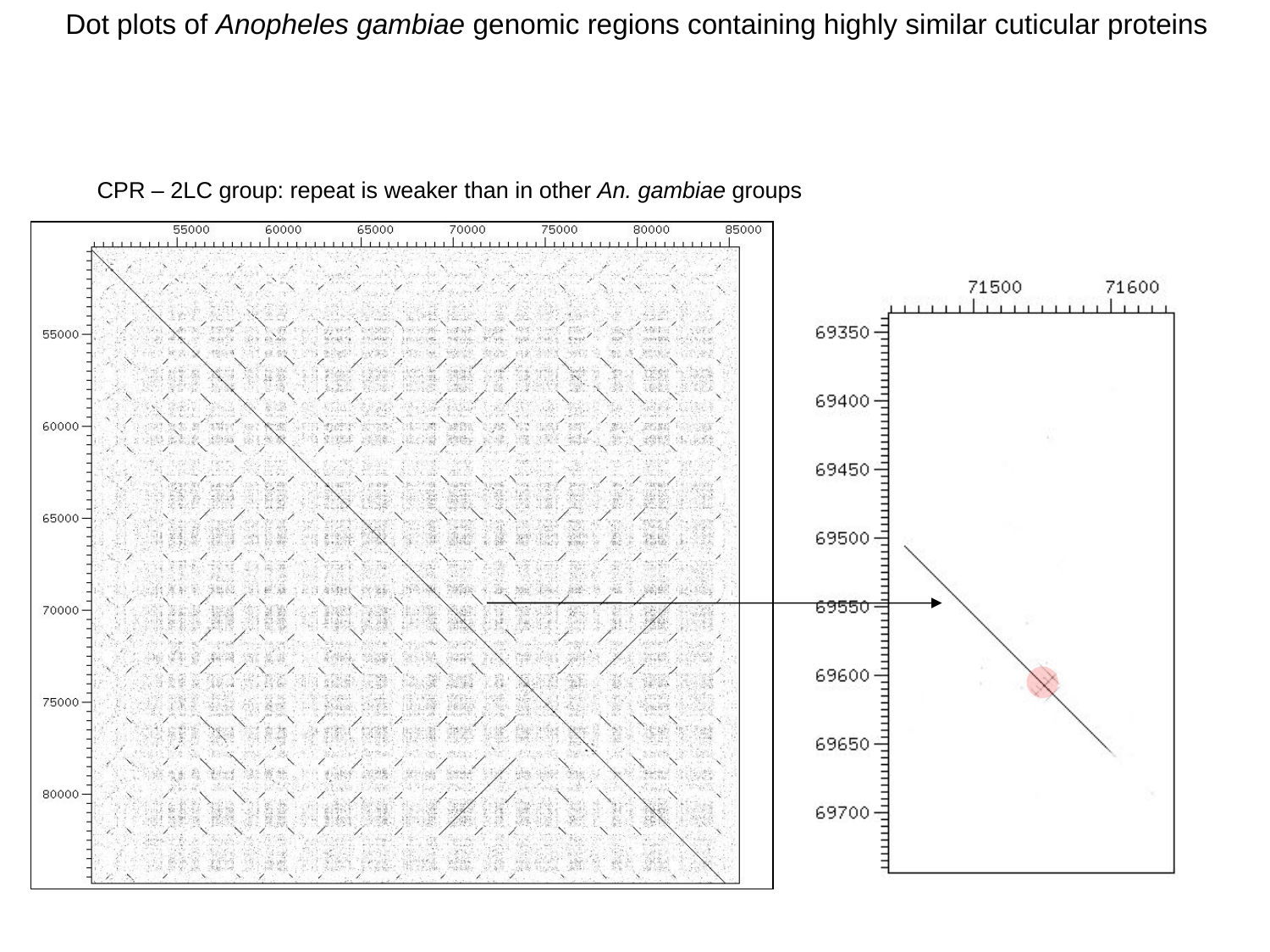

Dot plots of Anopheles gambiae genomic regions containing highly similar cuticular proteins
CPR – 2LC group: repeat is weaker than in other An. gambiae groups

## Slide 5
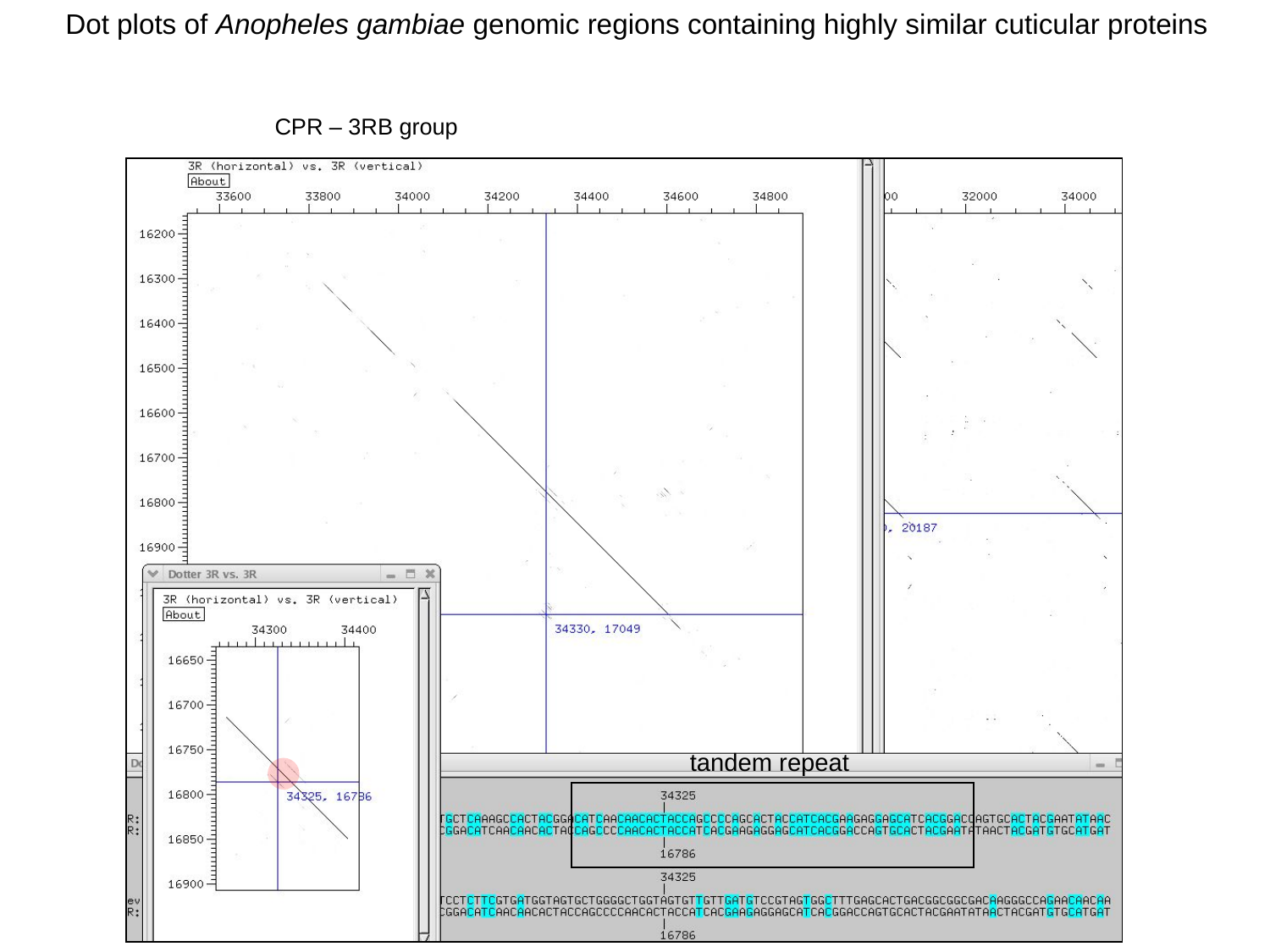

Dot plots of Anopheles gambiae genomic regions containing highly similar cuticular proteins
CPR – 3RB group
tandem repeat
